# Supplementary material for: Structural differences between the closely related RNA helicases, UAP56 and URH49, fashion distinct functional apo-complexes
Source: Nat Commun. 2024 Jan 15;15:455. doi: 10.1038/s41467-023-44217-8 (PMC10789772; doi:10.1038/s41467-023-44217-8)
Supplement: Supplementary file 1 — Supplementary Materials [file 41467_2023_44217_MOESM1_ESM.pdf]

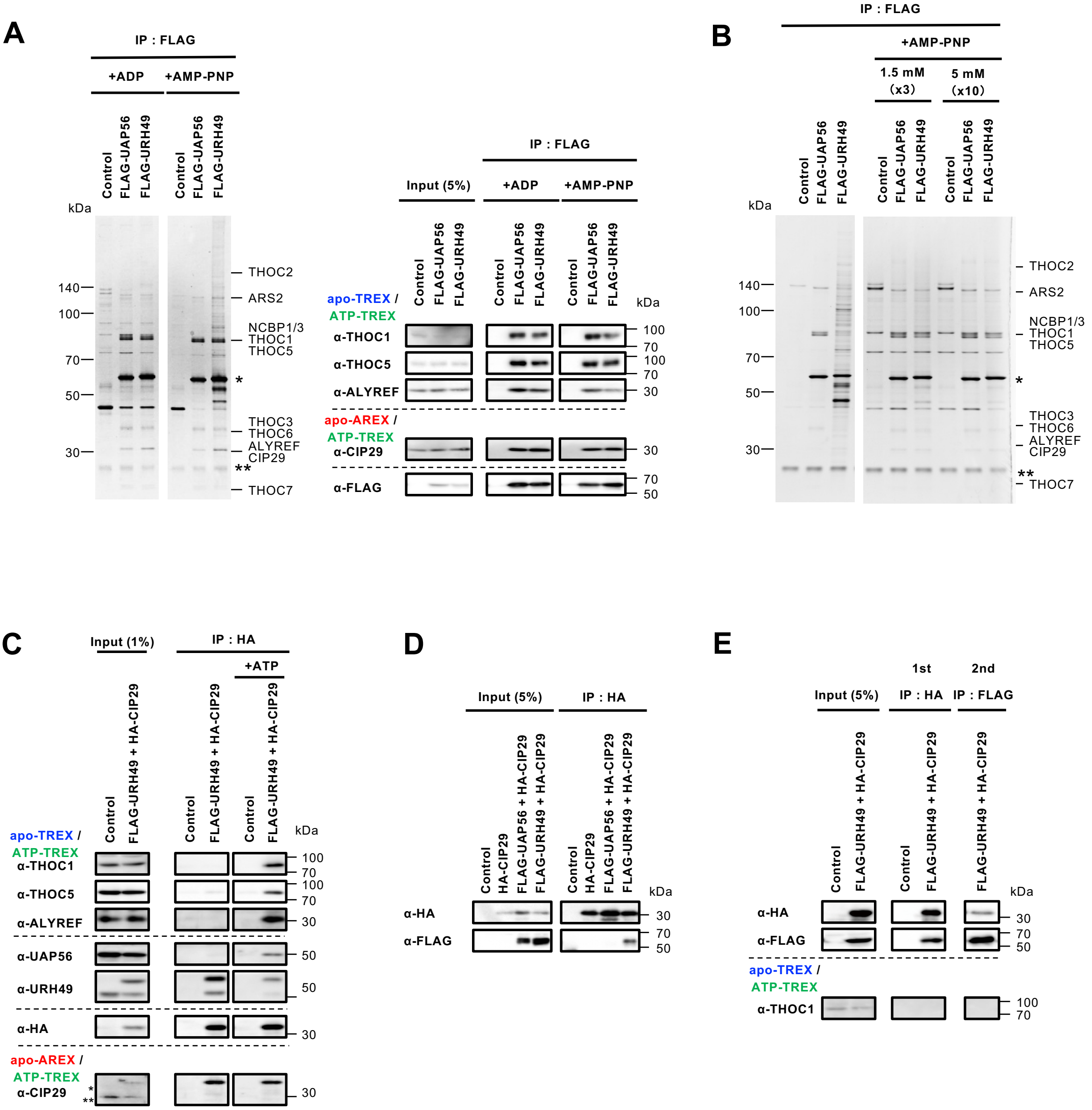
A
IP : FLAG
+ADP
+AMP-PNP
Control
FLAG-UAP56
FLAG-URH49
kDa
140
100
70
50
30
THOC2
ARS2
NCBP1/3
THOC1
THOC5
*
THOC3
THOC6
ALYREF
CIP29
**
THOC7
Input (5%)
apo-TREX /
ATP-TREX
α-THOC1
α-THOC5
α-ALYREF
apo-AREX /
ATP-TREX
α-CIP29
α-FLAG
B
1.5 mM (x3)
5 mM (x10)
C
Input (1%)
IP : HA
+ATP
FLAG-URH49 + HA-CIP29
α-UAP56
α-URH49
α-HA
D
HA-CIP29
FLAG-UAP56 + HA-CIP29
FLAG-URH49 + HA-CIP29
E
1st
2nd
IP : HA
IP : FLAG

Supplementary Fig.1. Novel apo-AREX candidates were identified by tandem-immunoprecipitation (Related to Fig.1)

Immunoprecipitation was performed using anti-DYKDDDDK tag antibody beads and HA antibody beads. Flp-In T-REx 293 cells stably expressing FLAG-tagged and/or HA-tagged protein were used to prepare nuclear extract. Precipitated samples were separated and detected by silver staining or immunoblotting with the indicated antibodies. **A** ADP or AMP-PNP addition promotes the remodeling of both the apo-AREX complex and apo-TREX complex to the ATP-TREX complex. Single asterisk represents precipitated FLAG-UAP56 or -URH49. Double asterisk represents IgG light chain. **B** Remodeling of the apo-AREX complex to the ATP-TREX complex by the addition of excess AMP-PNP. Single asterisks represent precipitated FLAG-UAP56 or -URH49. Double asterisks represent IgG light chains. **C** HA-CIP29 was associated with FLAG-URH49, a component of the apo-AREX complex, in the ATP-depleted condition and became associated with the ATP-TREX complex components in the presence of ATP. Single and double asterisks represent HA- and endogenous-CIP29, respectively. **D** HA-CIP29 is associated with FLAG-URH49 but not with FLAG-UAP56 in the ATP-depleted condition. **E** Tandem-immunoprecipitation was performed to highly purify novel apo-AREX complex components. THOC1, a component of the apo- and ATP-TREX was used as the negative control. For panels **A**-**E**, similar results were obtained in at least three independent experimental settings. Source data are provided as a Source Data file.

A

FLAG-URH49 HA-CIP29 Tandem-immunoprecipitation (IP)

Term	Count	P-Value
RNA splicing	22	3.10E-18
RNA splicing, via transesterification reactions with bulged adenosine as nucleophile	19	1.10E-16
mRNA splicing, via spliceosome	19	1.10E-16
RNA splicing, via transesterification reactions	19	1.30E-16
mRNA processing	21	1.50E-15
RNA processing	26	4.60E-15
mRNA metabolic process	21	1.10E-12
gene expression	46	4.70E-09
RNA metabolic process	39	9.00E-07
regulation of gene expression	37	1.70E-06

FLAG-URH49 IP

Term	Count	P-Value
RNA splicing	35	8.60E-20
mRNA processing	36	1.70E-18
mRNA metabolic process	38	3.20E-15
RNA splicing, via transesterification reactions with bulged adenosine as nucleophile	26	1.50E-14
mRNA splicing, via spliceosome	26	1.50E-14
RNA splicing, via transesterification reactions	26	2.00E-14
regulation of mRNA stability	19	1.00E-13
regulation of RNA stability	19	2.30E-13
posttranscriptional regulation of gene expression	30	2.90E-13
RNA processing	39	7.10E-12

FLAG-UAP56 IP

Term	Count	P-Value
RNA splicing	26	3.30E-18
mRNA processing	27	1.30E-17
nuclear export	19	1.20E-16
mRNA metabolic process	29	9.60E-16
mRNA transport	17	9.80E-16
viral mRNA export from host cell nucleus	8	2.80E-15
RNA transport	17	2.00E-14
nucleic acid transport	17	2.00E-14
establishment of RNA localization	17	2.60E-14
RNA export from nucleus	15	4.10E-14

B

GeneName	Tandem-IP	FLAG-URH49 IP	FLAG-UAP56 IP
DDX39A	736	1097	280
TUBA1C	490	678	206
CIP29	478	505	422
ILF3	396	147	0
TUBB	374	502	0
HNRNPH1	350	291	114
HNRNPUL1	319	0	0
TUBB4B	291	433	0
HNRNPM	272	523	0
HNRNPA0	270	176	102
DSP	238	3331	1520
EEF1A1P5	235	463	0
ILF2	229	108	0
HNRNPA3	206	16	65
DDX5	190	10	0
HNRNPA1	180	0	30
SFPQ	168	0	0
ACTB	160	300	52
C14orf166	157	0	0
FUS	150	73	107
DDX17	146	0	0
DDX1	138	0	0
HNRNPU	135	0	0
RUVBL2	125	473	0
RBMX	121	0	325
hCG_2039566	115	169	0
TUBA4B	112	113	0
ACTBL2	110	111	0
GFAP	99	33	0
HIST1H4A	91	241	159
DDX39B	88	105	1412
HNRNPR	82	0	0
CPVL	80	0	0
TXN	77	130	109
RTCB	76	0	0
FLG2	71	413	288
VIM	68	180	0
HNRNPAB	64	66	96
SBSN	64	603	0
SF3B6	55	0	0
CASP14	45	281	389
TFAP2D	45	0	0
DHX29	43	0	0
MEX3A	42	42	31
RUVBL1	42	490	0
TSPAN10	40	0	0
NLRC3	39	0	0
FAM25A	39	0	0
GAPDH	38	194	338
HIST1H2BN	38	314	86
ACTA1	37	37	37
ABCA5	37	0	0
TUFM	37	34	40
USP17L11	36	33	32
RHEB	36	38	0
CALD1	35	0	0
HRNR	35	29	1
HNRNPDL	34	0	0
CSTA	34	70	0
KHDRBS1	34	0	0
C3	33	0	0
KPRP	33	0	188
EEF1A1	32	220	0
ADAR	32	0	0
XP32	31	41	46
HNRNPL	20	0	0
HNRNPC	16	278	156
IGKV4-1	11	0	2
EWSR1	9	0	0
FLG	7	247	338
ALG13	4	6	0
ASAP2	4	0	0
LRMP	3	0	3
H3F3A	3	41	83
JUP	2	719	744
PPP6R2	1	0	0
HNRNPA2B1	1	25	65
DCD	1	0	78

Protein Score: Low – High (0, 100, 200, 300, 400, > 500)

C

GeneName	Tandem-IP	FLAG-URH49 IP	FLAG-UAP56 IP	Type
SNRPD3	0	57	52	Sm
DDX17	568	0	0	A
DDX5	405	10	0	A
FUS	136	73	107	A
HSPB1	0	105	0	B
JUP	0	719	744	C2
MATR3	32	119	45	C2
SF3A3	83	34	0	U2
CD2BP2	0	34	0	U5
PRPF6	0	34	0	U5
HSPA8	194	568	113	Prp19
PPIE	0	50	0	Prp19 rel
SRSF2	0	39	0	SR
SRSF3	0	53	0	SR
SRSF9	0	0	32	SR
HNRNPA0	216	176	102	hnRNP
HNRNPA1	547	0	30	hnRNP
HNRNPA2B1	193	25	65	hnRNP
HNRNPA3	483	16	65	hnRNP
HNRNPAB	229	66	96	hnRNP
HNRNPC	0	278	156	hnRNP
HNRNPD	129	101	0	hnRNP
HNRNPF	293	296	0	hnRNP
HNRNPH1	531	291	114	hnRNP
HNRNPH3	59	20	2	hnRNP
HNRNPM	66	523	0	hnRNP
HNRNPR	386	0	0	hnRNP
HNRNPU	158	0	0	hnRNP
HNRNPUL1	35	0	0	hnRNP
PCBP1	0	37	0	hnRNP
RALY	0	167	0	hnRNP
RBMX	128	0	325	hnRNP
BAG2	0	232	0	MISC
ILF3	452	147	0	MISC
KHDRBS1	226	0	0	MISC
H1F0	0	47	0	Histone
H2AFY	0	91	0	Histone
H2AFZ	0	114	0	Histone
H3F3A	70	41	83	Histone
H3F3B	0	207	0	Histone
hCG_2039566	103	169	0	Histone
HIST1H1B	0	140	0	Histone
HIST1H1E	0	219	0	Histone
HIST1H2AG	0	220	163	Histone
HIST1H2BN	71	314	86	Histone
HIST1H4A	0	241	159	Histone

Protein Score: Low – High (0, 100, 200, 300, 400, > 500)

D

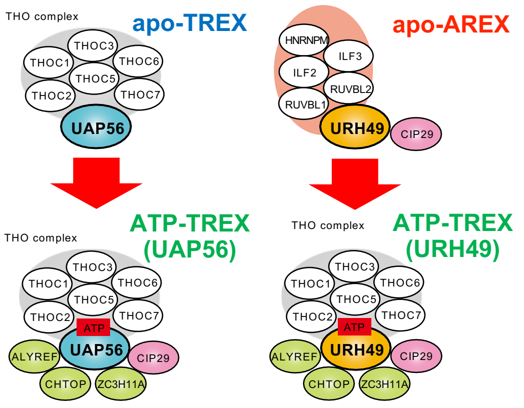

Supplementary Fig.2. The feature of proteins associated with UAP56 or URH49 in the ATP-depleted condition (Related to Fig.1)

A GO terms categorized in "Biological Process" for proteins identified by each immunoprecipitation. **B** Prot_scores of immunoprecipitations from tandem-immunoprecipitation precipitants (Tandem-IP), immunoprecipitations from FLAG-URH49 (FLAG-URH49 IP), and immunoprecipitations from FLAG-UAP56 (FLAG-UAP56 IP) were calculated by subtracting that from the control using Mascot software (Matrix Science). If multiple prot_scores were presented for one protein, the highest value was listed. Proteins with Prot_score > 0 in tandem-immunoprecipitation precipitants were described. Other factors which were detected in immunoprecipitations from FLAG-UAP56 or FLAG-URH49 were described in Supplementary Table 1. In the lower-left bar, each prot_score is indicated by the color intensity. **C** Spliceosome-associated factors were identified from each immunoprecipitation. **D** The model of the apo- and ATP-complex formation of UAP56 and URH49.

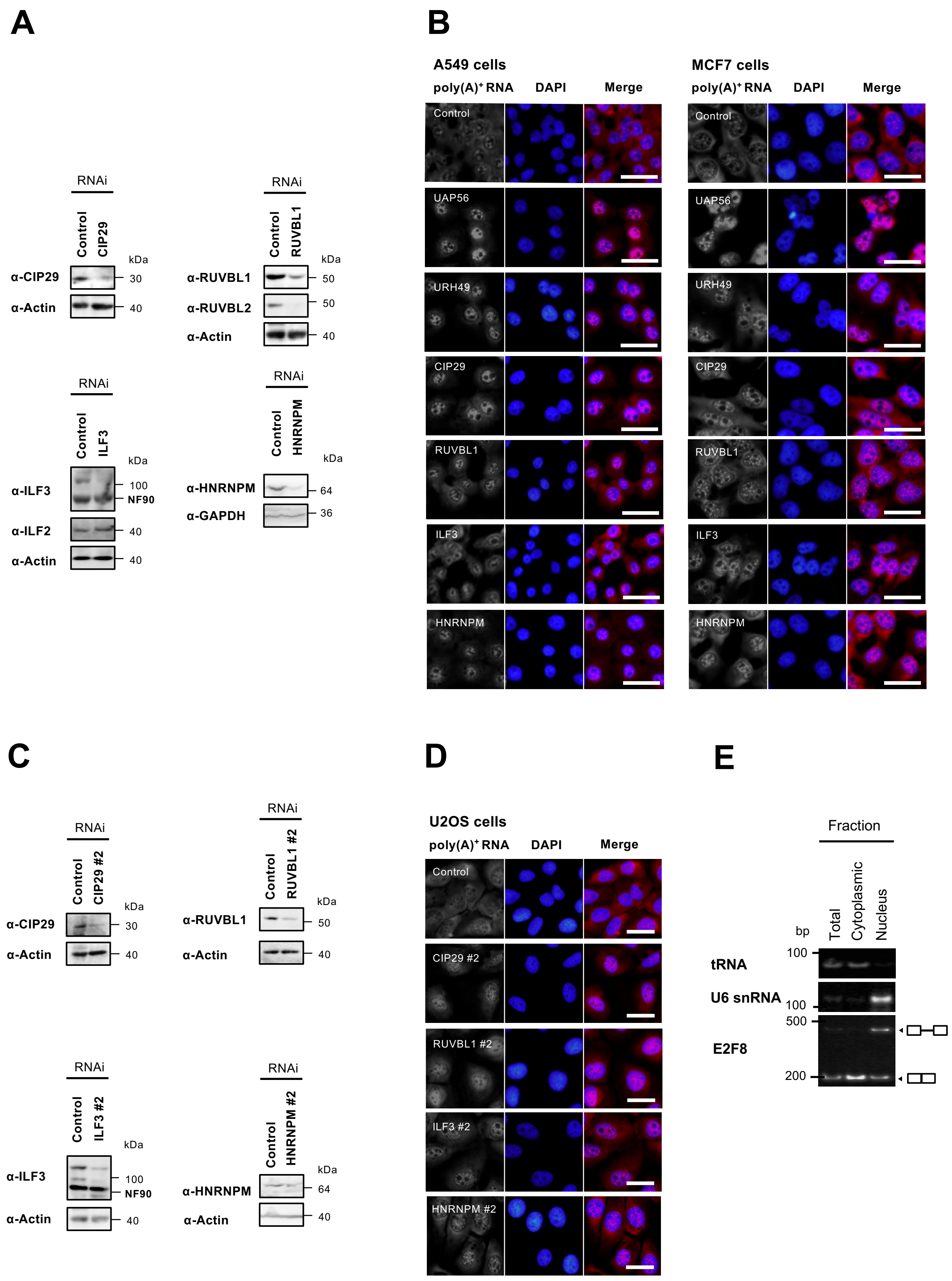
A
RNAi
Control
CIP29
kDa
α-CIP29
30
α-Actin
40
RNAi
Control
RUVBL1
kDa
α-RUVBL1
50
α-RUVBL2
50
α-Actin
40
RNAi
Control
ILF3
kDa
α-ILF3
100
NF90
α-ILF2
40
α-Actin
40
RNAi
Control
HNRNPM
kDa
α-HNRNPM
64
α-GAPDH
36
B
A549 cells
poly(A)+ RNA
DAPI
Merge
MCF7 cells
poly(A)+ RNA
DAPI
Merge
Control
UAP56
URH49
CIP29
RUVBL1
ILF3
HNRNPM
C
RNAi
Control
CIP29 #2
kDa
α-CIP29
30
α-Actin
40
RNAi
Control
RUVBL1 #2
kDa
α-RUVBL1
50
α-Actin
40
RNAi
Control
ILF3 #2
kDa
α-ILF3
100
NF90
α-Actin
40
RNAi
Control
HNRNPM #2
kDa
α-HNRNPM
64
α-Actin
40
D
U2OS cells
poly(A)+ RNA
DAPI
Merge
Control
CIP29 #2
RUVBL1 #2
ILF3 #2
HNRNPM #2
E
Fraction
bp
Total
Cytoplasmic
Nucleus
tRNA
100
U6 snRNA
100
E2F8
500
200

Supplementary Fig.3. Apo-AREX components function in the mRNA processing and export process (Related to Fig.2)

A Knockdown efficiency of an individual protein in U2OS cells was validated by immunoblotting. Actin or GAPDH was used as a loading control. **B** Depletion of apo-AREX components caused nuclear poly $(A)^+$ RNA accumulation in A549 cells and MCF7 cells. DAPI was used to visualize the nuclei. Scale bar, 40 μm. **C** Knockdown efficiency of an individual protein in U2OS cells using another siRNA against different sequence was estimated by immunoblotting. Actin was used as a loading control. **D** Nuclear poly $(A)^+$ RNA accumulation caused by siRNA treatment against apo-AREX components in U2OS cells. DAPI was used to visualize the nuclei. Scale bar, 40 μm. **E** The fractionations of the nuclear and the cytoplasmic RNA were examined by RT-PCR. tRNA served as a cytoplasmic marker and U6 snRNA was used as a nuclear marker. The E2F8 pre-mRNA predominantly localizes to the nucleus, whereas its spliced mRNA localizes to the cytoplasm. Thus, E2F8 was used to confirm the successful fractionation. DNA size in base pairs (bp) is indicated on the left side. For panels **A**-**E**, similar results were obtained in at least three independent experimental settings. Source data are provided as a Source Data file.

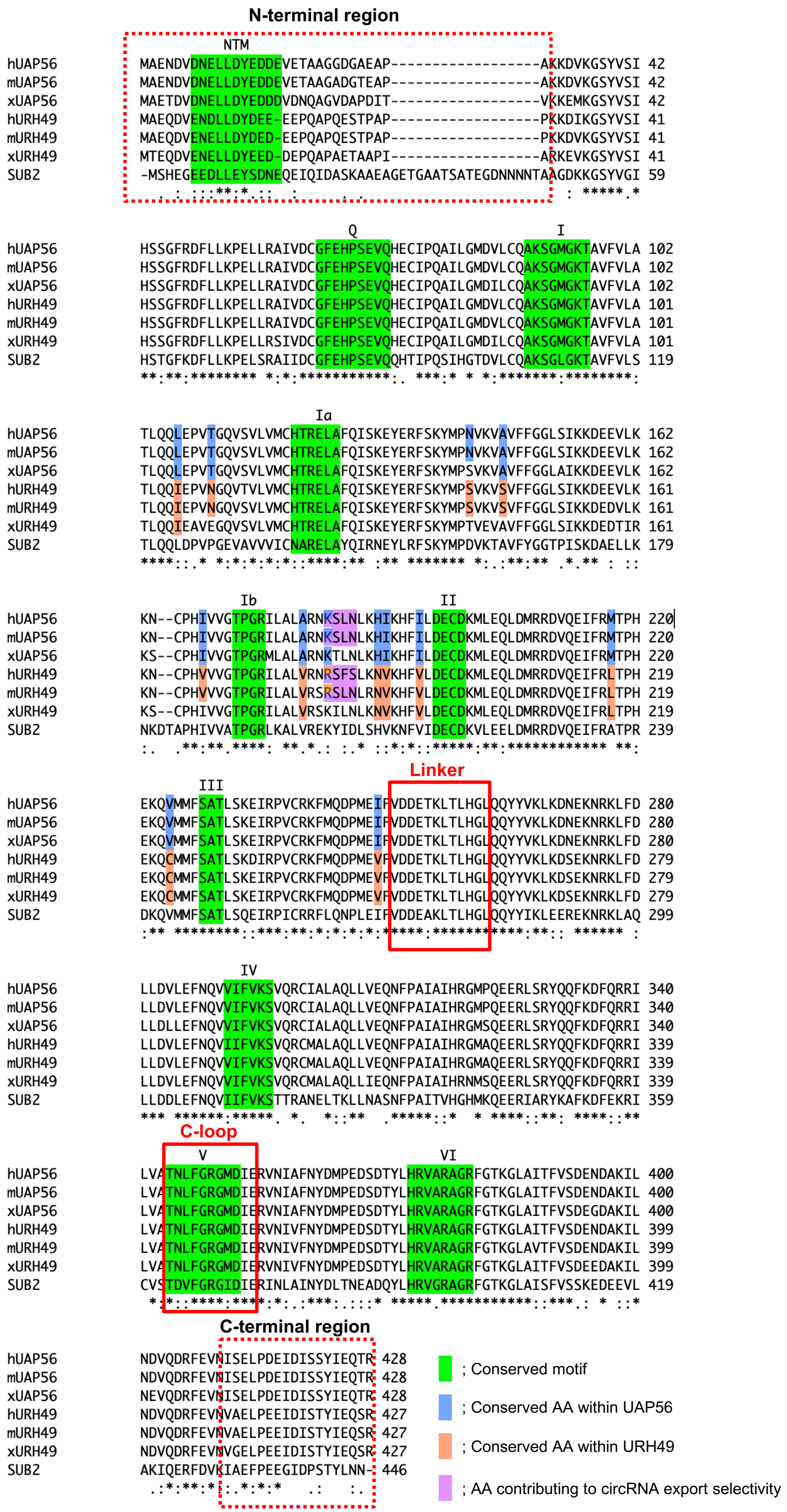
N-terminal region
NTM
hUAP56 MAENDVDNELLDYEDDEVETAAGGDGAEAP------------------AKKDVKGSYVSI 42
mUAP56 MAENDVDNELLDYEDDEVETAAGADGTEAP------------------AKKDVKGSYVSI 42
xUAP56 MAETDVDNELLDYEDDDVDNQAGVDAPDIT------------------VKKEMKGSYVSI 42
hURH49 MAEQDVENDLLDYDEE-EEPQAPQESTPAP------------------PKKDIKGSYVSI 41
mURH49 MAEQDVENELLDYDED-EEPQAPQESTPAP------------------PKKDVKGSYVSI 41
xURH49 MTEQDVENELLDYEED-DEPQAPAETAAPI------------------ARKEVKGSYVSI 41
SUB2 -MSHEGEEDLLEYSDNEQEIQIDASKAAEAGETGAATSATEGDNNNNTAAGDKKGSYVGI 59
Q
I
hUAP56 HSSGFRDFLLKPELLRAIVDCGFEHPSEVQHECIPQAILGMDVLCQAKSGMGKTAVFVLA 102
mUAP56 HSSGFRDFLLKPELLRAIVDCGFEHPSEVQHECIPQAILGMDVLCQAKSGMGKTAVFVLA 102
xUAP56 HSSGFRDFLLKPELLRAIVDCGFEHPSEVQHECIPQAILGMDILCQAKSGMGKTAVFVLA 102
hURH49 HSSGFRDFLLKPELLRAIVDCGFEHPSEVQHECIPQAILGMDVLCQAKSGMGKTAVFVLA 101
mURH49 HSSGFRDFLLKPELLRAIVDCGFEHPSEVQHECIPQAILGMDVLCQAKSGMGKTAVFVLA 101
xURH49 HSSGFRDFLLKPELLRSIVDCGFEHPSEVQHECIPQAILGMDILCQAKSGMGKTAVFVLA 101
SUB2 HSTGFKDFLLKPELSRAIIDCGFEHPSEVQQHTIPQSIHGTDVLCQAKSGLGKTAVFVLS 119
Ia
hUAP56 TLQQLEPVTGQVSVLVMCHTRELAFQISKEYERFSKYMPNVKVAVFFGGLSIKKDEEVLK 162
mUAP56 TLQQLEPVTGQVSVLVMCHTRELAFQISKEYERFSKYMPNVKVAVFFGGLSIKKDEEVLK 162
xUAP56 TLQQLEPVTGQVSVLVMCHTRELAFQISKEYERFSKYMPSVKVAVFFGGLAIKKDEEVLK 162
hURH49 TLQQIEPVNGQVTVLVMCHTRELAFQISKEYERFSKYMPSVKVSVFFGGLSIKKDEEVLK 161
mURH49 TLQQIEPVNGQVSVLVMCHTRELAFQISKEYERFSKYMPSVKVSVFFGGLSIKKDEDVLK 161
xURH49 TLQQIEAVEGQVSVLVMCHTRELAFQISKEYERFSKYMPTVEVAVFFGGLSIKKDEDTIR 161
SUB2 TLQQLDPVPGEVAVVVICNARELAYQIRNEYLRFSKYMPDVKTAVFYGGTPISKDAELLK 179
Ib
II
hUAP56 KN--CPHIVVGTPGRILALARNKSLNLKHIKHFILDECDKMLEQLDMRRDVQEIFRMTPH 220
mUAP56 KN--CPHIVVGTPGRILALARNKSLNLKHIKHFILDECDKMLEQLDMRRDVQEIFRMTPH 220
xUAP56 KS--CPHIVVGTPGRMLALARNKTLNLKHIKHFILDECDKMLEQLDMRRDVQEIFRMTPH 220
hURH49 KN--CPHVVVGTPGRILALVRNRSFSLKNVKHFVLDECDKMLEQLDMRRDVQEIFRLTPH 219
mURH49 KN--CPHVVVGTPGRILALVRSRSLNLRNVKHFVLDECDKMLEQLDMRRDVQEIFRLTPH 219
xURH49 KS--CPHIVVGTPGRILALVRSKILNLKNVKHFVLDECDKMLEQLDMRRDVQEIFRLTPH 219
SUB2 NKDTAPHIVVATPGRLKALVREKYIDLSHVKNFVIDECDKVLEELDMRRDVQEIFRATPR 239
Linker
III
hUAP56 EKQVMMFSATLSKEIRPVCRKFMQDPMEIFVDDETKLTLHGLQQYYVKLKDNEKNRKLFD 280
mUAP56 EKQVMMFSATLSKEIRPVCRKFMQDPMEIFVDDETKLTLHGLQQYYVKLKDNEKNRKLFD 280
xUAP56 EKQVMMFSATLSKEIRPVCRKFMQDPMEIFVDDETKLTLHGLQQYYVKLKDNEKNRKLFD 280
hURH49 EKQCMMFSATLSKDIRPVCRKFMQDPMEVFVDDETKLTLHGLQQYYVKLKDSEKNRKLFD 279
mURH49 EKQCMMFSATLSKEIRPVCRKFMQDPMEVFVDDETKLTLHGLQQYYVKLKDSEKNRKLFD 279
xURH49 EKQCMMFSATLSKEIRPVCRKFMQDPMEVFVDDETKLTLHGLQQYYVKLKDSEKNRKLFD 279
SUB2 DKQVMMFSATLSQEIRPICRRFLQNPLEIFVDDEAKLTLHGLQQYYIKLEEREKNRKLAQ 299
IV
hUAP56 LLDVLEFNQVVIFVKSVQRCIALAQLLVEQNFPAIAIHRGMPQEERLSRYQQFKDFQRRI 340
mUAP56 LLDVLEFNQVVIFVKSVQRCIALAQLLVEQNFPAIAIHRGMPQEERLSRYQQFKDFQRRI 340
xUAP56 LLDLLEFNQVVIFVKSVQRCIALAQLLVEQNFPAIAIHRGMSQEERLSRYQQFKDFQRRI 340
hURH49 LLDVLEFNQVIIFVKSVQRCMALAQLLVEQNFPAIAIHRGMAQEERLSRYQQFKDFQRRI 339
mURH49 LLDVLEFNQVVIFVKSVQRCMALAQLLVEQNFPAIAIHRGMAQEERLSRYQQFKDFQRRI 339
xURH49 LLDVLEFNQVVIFVKSVQRCMALAQLLIEQNFPAIAIHRNMSQEERLSRYQQFKDFQRRI 339
SUB2 LLDDLEFNQVIIFVKSTTRANELTKLLNASNFPAITVHGHMKQEERIARYKAFKDFEKRI 359
C-loop
V
VI
hUAP56 LVATNLFGRGMDIERVNIAFNYDMPEDSDTYLHRVARAGRFGTKGLAITFVSDENDAKIL 400
mUAP56 LVATNLFGRGMDIERVNIAFNYDMPEDSDTYLHRVARAGRFGTKGLAITFVSDENDAKIL 400
xUAP56 LVATNLFGRGMDIERVNIAFNYDMPEDSDTYLHRVARAGRFGTKGLAITFVSDEGDAKIL 400
hURH49 LVATNLFGRGMDIERVNIVFNYDMPEDSDTYLHRVARAGRFGTKGLAITFVSDENDAKIL 399
mURH49 LVATNLFGRGMDIERVNIVFNYDMPEDSDTYLHRVARAGRFGTKGLAVTFVSDENDAKIL 399
xURH49 LVATNLFGRGMDIERVNIVFNYDMPEDSDTYLHRVARAGRFGTKGLAITFVSDEEDAKIL 399
SUB2 CVSTDVFGRGIDIERINLAINYDLTNEADQYLHRVGRAGRFGTKGLAISFVSSKEDEEVL 419
C-terminal region
hUAP56 NDVQDRFEVNISELPDEIDISSYIEQTR 428
mUAP56 NDVQDRFEVNISELPDEIDISSYIEQTR 428
xUAP56 NEVQDRFEVNISELPDEIDISSYIEQTR 428
hURH49 NDVQDRFEVNVAELPEEIDISTYIEQSR 427
mURH49 NDVQDRFEVNVAELPEEIDISTYIEQSR 427
xURH49 NDVQDRFEVNVGELPEEIDISTYIEQSR 427
SUB2 AKIQERFDVKIAEFPEEGIDPSTYLNN- 446
; Conserved motif
; Conserved AA within UAP56
; Conserved AA within URH49
; AA contributing to circRNA export selectivity

Supplementary Fig.4. The alignment of UAP56 and URH49 orthologs (Related to Fig.3)

The prefix " h" indicates human, " m" indicates mouse, and " x" indicates Xenopus laevis. Conserved motifs of the DEAD-box helicase family are shown in green, as described previously[31]. The conserved amino acids in the N-domain of UAP56 or URH49 analyzed in this study are shown in blue or orange. The 4 amino acids that determines circular RNA export selectivity between UAP56 and URH49, are shown in purple[29].

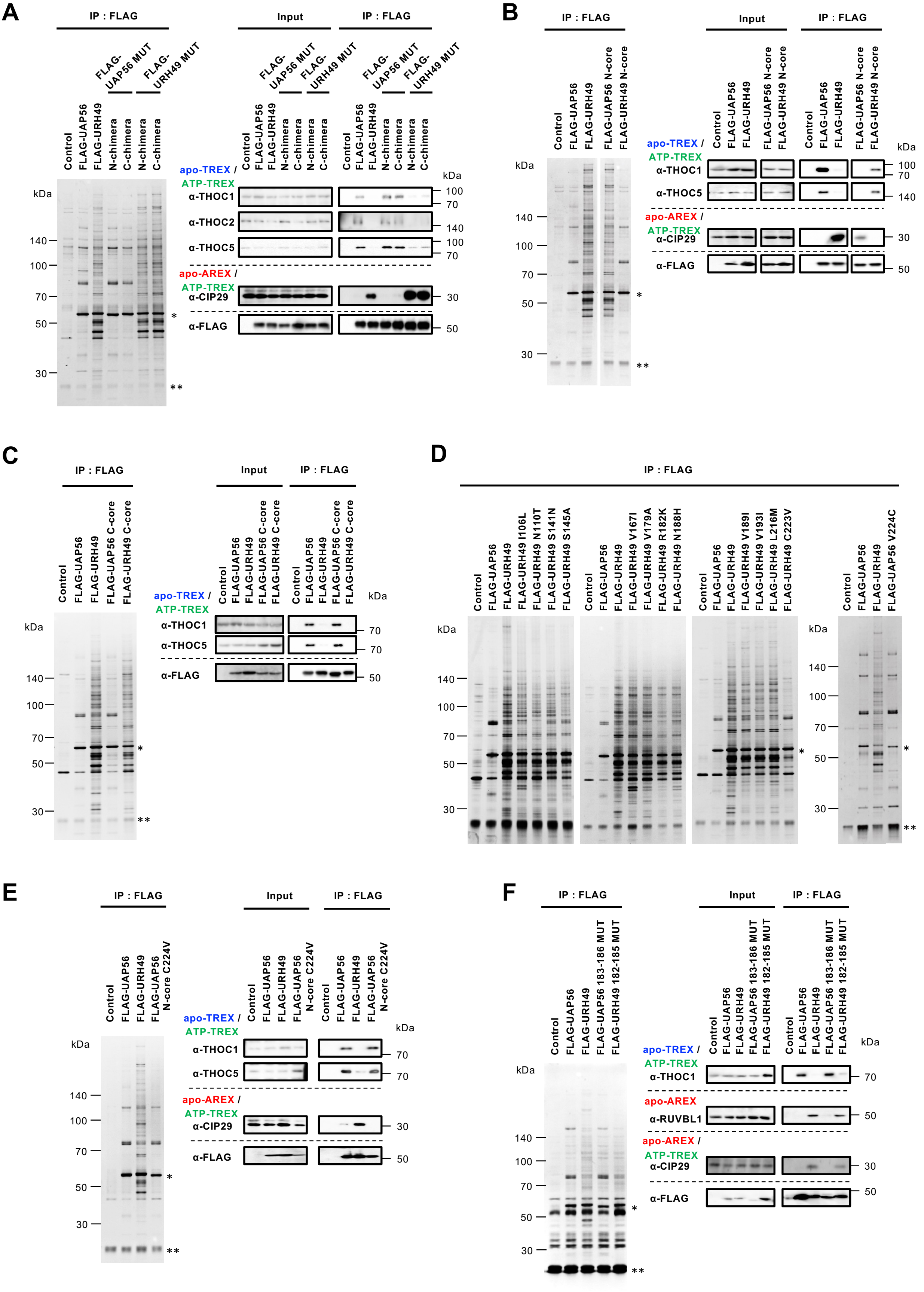
A
IP : FLAG
Input
IP : FLAG
FLAG-UAP56 MUT
FLAG-URH49 MUT
Control
FLAG-UAP56
FLAG-URH49
N-chimera
C-chimera
N-chimera
C-chimera
kDa
140
100
70
50
30
*
**
apo-TREX /
ATP-TREX
α-THOC1
α-THOC2
α-THOC5
apo-AREX /
ATP-TREX
α-CIP29
α-FLAG
100
70
140
100
70
30
50
B
IP : FLAG
Input
IP : FLAG
Control
FLAG-UAP56
FLAG-URH49
FLAG-UAP56 N-core
FLAG-URH49 N-core
kDa
140
100
70
50
30
*
**
apo-TREX /
ATP-TREX
α-THOC1
α-THOC5
apo-AREX /
ATP-TREX
α-CIP29
α-FLAG
100
70
140
30
50
C
IP : FLAG
Input
IP : FLAG
Control
FLAG-UAP56
FLAG-URH49
FLAG-UAP56 C-core
FLAG-URH49 C-core
kDa
140
100
70
50
30
*
**
apo-TREX /
ATP-TREX
α-THOC1
α-THOC5
α-FLAG
70
70
50
D
IP : FLAG
Control
FLAG-UAP56
FLAG-URH49
FLAG-URH49 I106L
FLAG-URH49 N110T
FLAG-URH49 S141N
FLAG-URH49 S145A
FLAG-URH49 V167I
FLAG-URH49 V179A
FLAG-URH49 R182K
FLAG-URH49 N188H
FLAG-URH49 V189I
FLAG-URH49 V193I
FLAG-URH49 L216M
FLAG-URH49 C223V
FLAG-UAP56 V224C
kDa
140
100
70
50
30
*
**
E
IP : FLAG
Input
IP : FLAG
Control
FLAG-UAP56
FLAG-URH49
FLAG-UAP56 N-core C224V
kDa
140
100
70
50
30
*
**
apo-TREX /
ATP-TREX
α-THOC1
α-THOC5
apo-AREX /
ATP-TREX
α-CIP29
α-FLAG
70
70
30
50
F
IP : FLAG
Input
IP : FLAG
Control
FLAG-UAP56
FLAG-URH49
FLAG-UAP56 183-186 MUT
FLAG-URH49 182-185 MUT
kDa
140
100
70
50
30
*
**
apo-TREX /
ATP-TREX
α-THOC1
apo-AREX
α-RUVBL1
apo-AREX /
ATP-TREX
α-CIP29
α-FLAG
70
50
30
50

Supplementary Fig.5. Complex formation by chimeric mutants (Related to Fig.3) Immunoprecipitations were performed using anti-DYKDDDDK tag antibody beads. Each precipitated sample was separated and detected by silver staining or immunoblotting with the indicated antibodies. Flp-In T-REx 293 cells stably expressing FLAG-tagged protein were used to prepare nuclear extract. **A-F**, Single asterisk represents precipitated FLAG-UAP56, -URH49 or -their mutants. Double asterisk represents IgG light chain. **A** N- and C-terminal regions of UAP56 and URH49 are not the determinant of the apo-TREX or the apo-AREX complex formation. **B** The N-domain of UAP56 and URH49 is essential for apo-complex formation. **C** The C-domain of UAP56 and URH49 is not the determinant of the apo-TREX or the apo-AREX complex formation. **D** The apo-complex formation of URH49 point mutants in the N-domain and UAP56 V224C, a point mutant of UAP56 in the N-domains. **E** The UAP56 N-core C224V mutant lost the ability to form the apo-AREX complex but did form the apo-TREX complex. The UAP56 Ncore C224V mutant is the UAP56 mutant in which the N-domains of UAP56 other than UAP56-V224 were replaced with the N-domains of URH49. **F** The apo-complex formation of chimeric mutants, in which the region responsible for the selection of circular RNAs in UAP56 and URH49 are swapped, did not affect the apo-TREX or the apo-AREX complex formation. For panels **A**-**F**, similar results were obtained in at least three independent experimental settings. Source data are provided as a Source Data file.

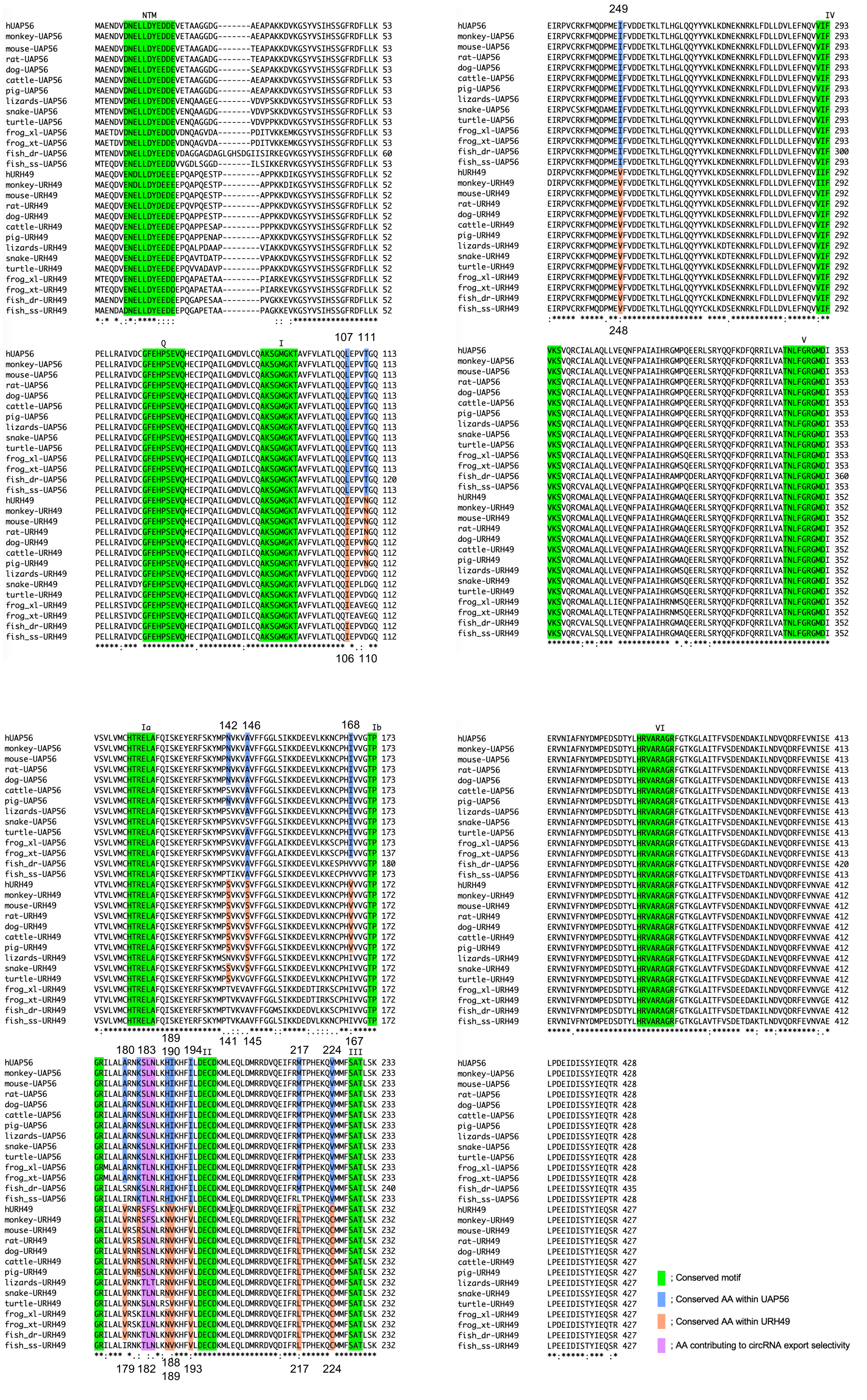

NTM
Q
I
Ia
Ib
II
III
IV
V
VI
; Conserved motif
; Conserved AA within UAP56
; Conserved AA within URH49
; AA contributing to circRNA export selectivity

Supplementary Fig.6. The extended alignment of UAP56 and URH49 orthologs in vertebrates (Related to Fig.3)

We selected 10 species of vertebrates and aligned the amino acid sequences of UAP56 and URH49. The scientific (Latin) names of each species analyzed are as follows: Monkey: Macaca mulatta, mouse: Mus musculus, rat: Rattus norvegicus, dog: Canis lupus familiaris, cattle: Bos taurus, pig: Sus scrofa, lizard: Pogona vitticeps, snake: Euleptes europaea, turtle: Dermochelys coriacea, frog_xl: Xenopus laevis, frog_xt: Xenopus tropicalis, fish_dr: Danio rerio, fish_ss: Salmo salar. Conserved motifs of the DEAD-box helicase family are shown in green as described previously[31]. Conserved amino acids in the N-domains of UAP56 and URH49 analyzed in this study are shown in blue and orange, respectively. The four amino acids that determine circular RNA export selectivity between UAP56 and URH49 are shown in purple[29].

	monkey	mouse	rat	dog	cattle	pig	lizards	snake	turtle	frog_xl	frog_xt	fish_dr	fish_ss
hUAP56 107L													
hUAP56 111T													
hUAP56 142N					S		S	S	S	S	S	S	T
hUAP56 146A								S					
hUAP56 168I												V	V
hUAP56 180A												S	I
hUAP56 183K													
hUAP56 189H													
hUAP56 190I													
hUAP56 194I													
hUAP56 217M													L
hUAP56 224V													
hUAP56 249I													

	monkey	mouse	rat	dog	cattle	pig	lizards	snake	turtle	frog_xl	frog_xt	fish_dr	fish_ss
hURH49 106I											T		
hURH49 110N							D	D	D	E	E	D	D
hURH49 141S							N			T	T	T	T
hURH49 145S									G	A	A	A	A
hURH49 167V							I	I	I	I	I	I	I
hURH49 179V													I
hURH49 182R							K	K	K	K	K	K	K
hURH49 188N									S				
hURH49 189V													
hURH49 193V													
hURH49 216L													
hURH49 223C													
hURH49 248V													

; UAP56-type AA

; URH49-type AA

Supplementary Fig.7. The conserved amino acid sequence in the alignment of UAP56 and URH49 orthologs (Related to Fig.3)

This panel shows the 12 amino acids in the N-domain of UAP56 or URH49 from each species compared with those of human UAP56 or URH49 analyzed in this study. Amino acids of the UAP56-type are shown in blue and those of the URH49-type in orange. An amino acid that differed from the corresponding amino acid in human UAP56 or URH49 in each species was indicated.

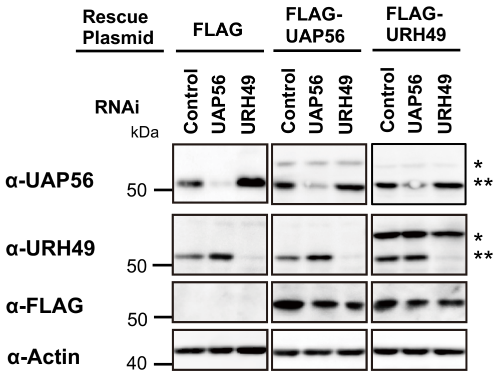
Rescue Plasmid
FLAG
FLAG-UAP56
FLAG-URH49
RNAi
Control
UAP56
URH49
Control
UAP56
URH49
Control
UAP56
URH49
kDa
α-UAP56
50
α-URH49
50
α-FLAG
50
α-Actin
40
*
**
*
**

Supplementary Fig.8. Forced expression of siRNA-resistant FLAG-UAP56 or URH49 in the endogenous UAP56 or URH49 depleted condition (Related to Fig.3) Single and double asterisks represent FLAG- and endogenous-UAP56 and URH49, respectively. Each precipitated sample was separated and detected by immunoblotting with the indicated antibodies. Similar results were obtained in at least three independent experimental settings. Source data are provided as a Source Data file.

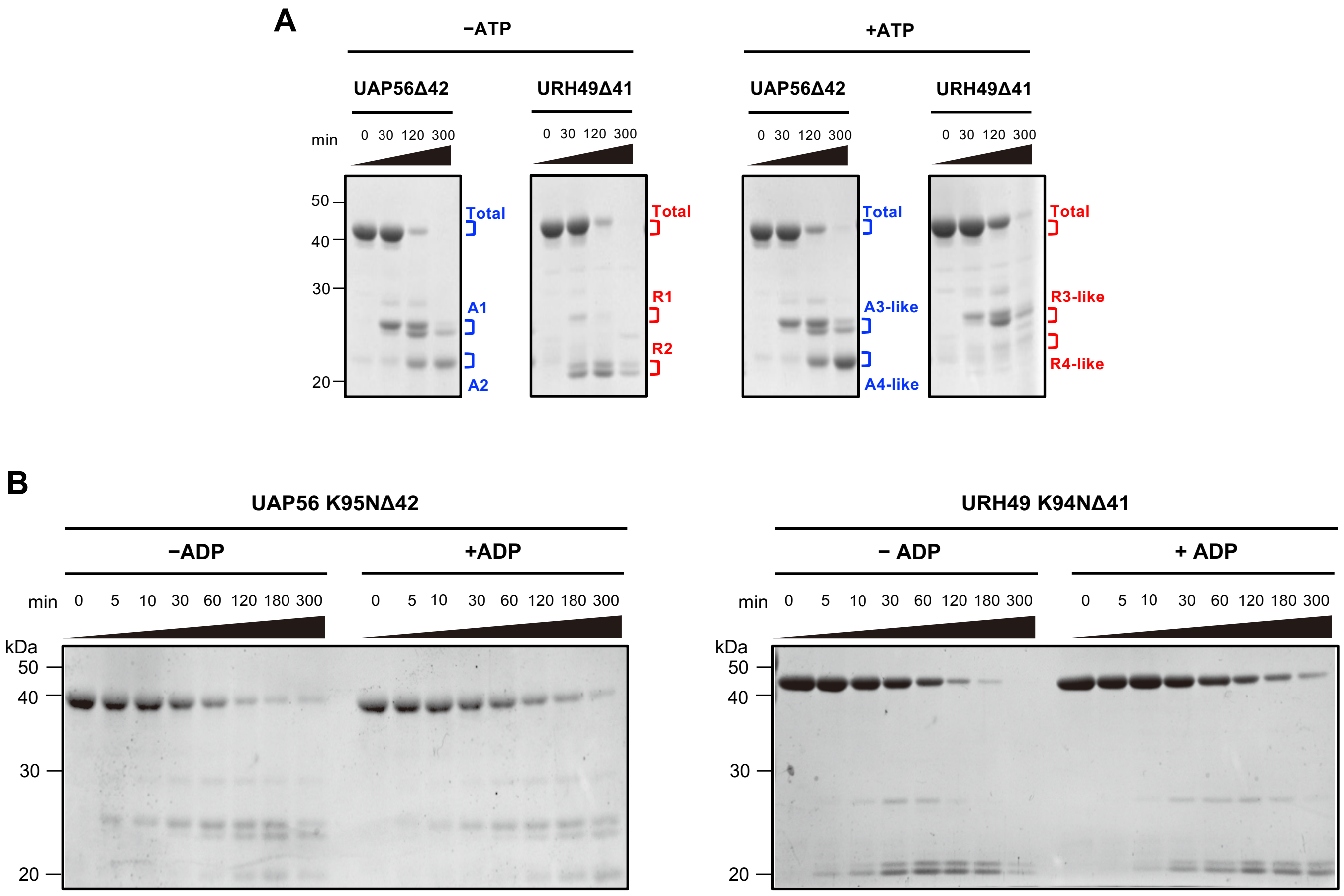
A
−ATP
+ATP
UAP56Δ42
URH49Δ41
UAP56Δ42
URH49Δ41
min
0 30 120 300
50
40
30
20
Total
A1
A2
R1
R2
A3-like
A4-like
R3-like
R4-like
B
UAP56 K95NΔ42
URH49 K94NΔ41
−ADP
+ADP
− ADP
+ ADP
min
0 5 10 30 60 120 180 300
kDa
50
40
30
20

Supplementary Fig.9. The partial digestion patterns of UAP56Δ42 and URH49Δ41 (Related to Fig.4)

Purified proteins were partially digested with trypsin. Aliquots were collected at each time point, separated by SDS-PAGE, and detected by Coomassie staining. **A** The limited proteolysis patterns of UAP56Δ42 and URH49Δ41 in the presence of ATP closely resembled those obtained when ADP was added. **B** ATP-binding DEAD mutants, UAP56 K95NΔ42 and URH49 K94NΔ41 did not change their digestion patterns in the absence and the presence of ADP. For panels **A** and **B**, similar results were obtained in at least three independent experimental settings. Source data are provided as a Source Data file.

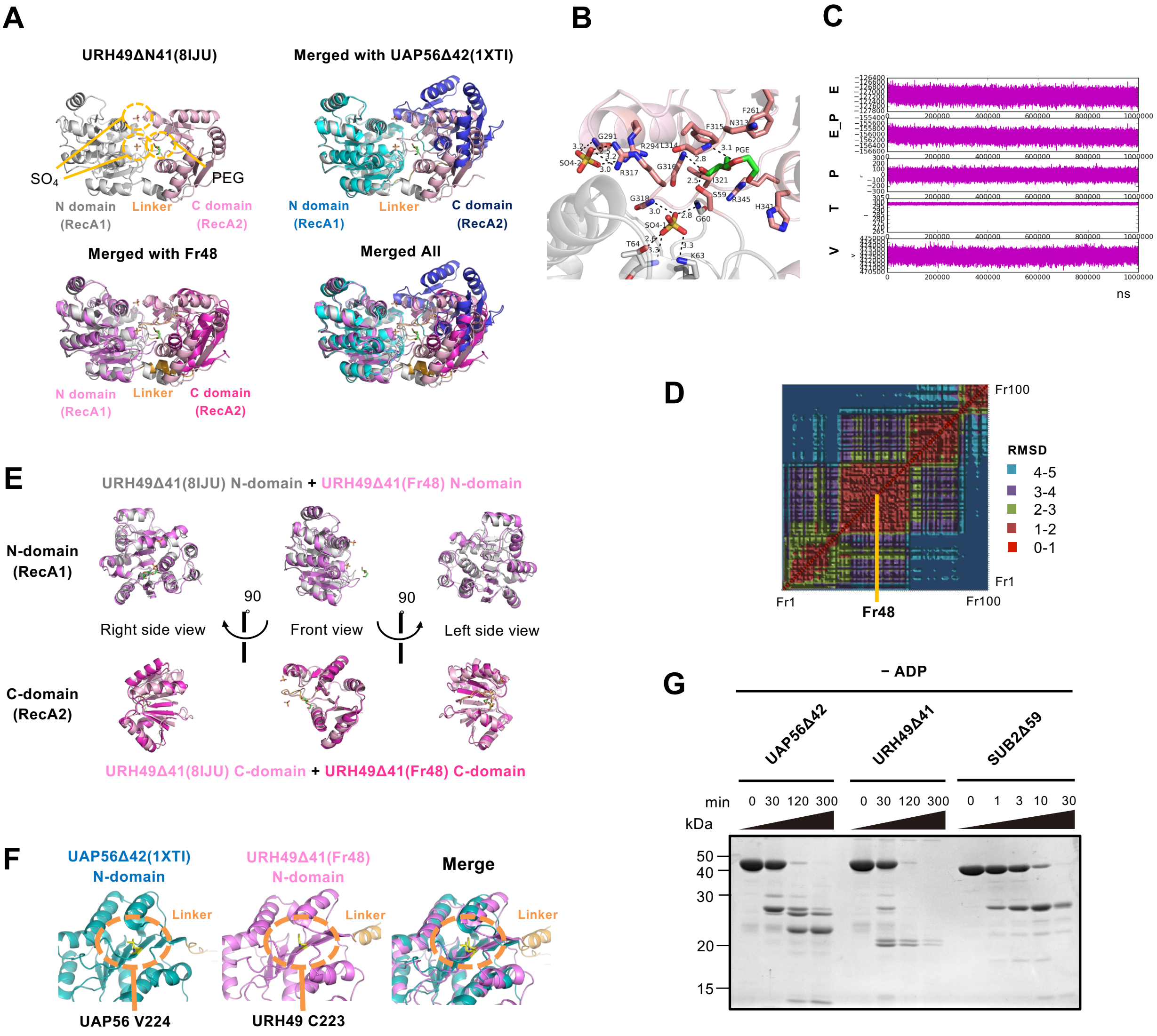
A
URH49ΔN41(8IJU)
Merged with UAP56Δ42(1XTI)
SO4
PEG
N domain (RecA1)
Linker
C domain (RecA2)
Merged with Fr48
Merged All
B
C
E
E_P
P
T
V
ns
D
Fr100
Fr1
Fr48
RMSD
4-5
3-4
2-3
1-2
0-1
E
URH49Δ41(8IJU) N-domain + URH49Δ41(Fr48) N-domain
N-domain (RecA1)
C-domain (RecA2)
90
Right side view
Front view
Left side view
URH49Δ41(8IJU) C-domain + URH49Δ41(Fr48) C-domain
F
UAP56Δ42(1XTI) N-domain
URH49Δ41(Fr48) N-domain
Merge
UAP56 V224
URH49 C223
G
− ADP
UAP56Δ42
URH49Δ41
SUB2Δ59
min
0 30 120 300
0 1 3 10 30
kDa
50
40
30
20
15

Supplementary Fig.10. Construction of URH49ΔN41 apo-structure models and comparisons of enzyme activities and structural features between UAP56Δ42, URH49Δ41 and Sub2Δ59 (Related to Fig.5)

A Comparison of apo-UAP56Δ42 crystal structure (1XTI), URH49Δ41 crystal structure (8IJU), and URH49Δ41 apo-structure model (Fr48). **B** Coordination of SO_4^{2-} in the ATP binding pocket of the URH49Δ41 crystal structure. **C** The time course of thermodynamic parameters during MD simulation of apo-URH49Δ41. Each calculated thermodynamic score is shown. E ; total energy, E_P ; potential energy, P ; pressure, T ; cluster temperature, and V ; volume, respectively. **D** 2D-RMSD matrix of MD trajectories of the apo-URH49 Δ41. The RMSDs of each apo-URH49Δ41 structural model were calculated and classified into 6 clusters based on the differences. Among the representative frames in the clusters, an apo-structure model, Fr48, located in the center of the largest central cluster, was treated as the apo-URH49Δ41 representative model structure. **E** Comparison of N-domain and C-domain between URH49Δ41 crystal (8IJU) and URH49Δ41 model structure (Fr48). **F** Three-dimensional arrangement of UAP56-V223 and URH49-C223. **G** The apo-Sub2Δ59 exhibited a similar limited proteolysis pattern with the apo-UAP56Δ42 but not with the apo-URH49Δ41. Similar result was obtained in at least three independent experimental settings. Source data are provided as a Source Data file.

Supplementary Table 1 Detected protein list by immunoprecipitation from FLAG-UAP56 or URH49 followed by LC-MS/MS (Related to Fig.1 and Fig.S2)
Precipitate protein list identified by datasets containing precipitated proteins in immunoprecipitation of FLAG-UAP56 or -URH49 in the absence of ATP.

Supplementary Table 2 List of detected peptide fragment by limited proteolysis from UAP56ΔN42 or URH49ΔN41 followed by LC-MS/MS (Related to Fig. 4)

Supplementary Table 3 List of amino acid residues with significantly different conformations (Related to Fig. 5)

Supplementary Table 4 Antibodies list in this study

Supplementary Table 5 Primer list for plasmid construction used in this study

Supplementary Table 6 siRNA list used in this study

Supplementary Table 7 Primer list for RT-qPCR and RT-PCR used in this study

Supplementary Table 1. Detected protein list by immunoprecipitation from FLAG-UAP56 or URH49 followed by LC-MS/MS

prot_acc	GeneName	prot_mass	FLAG-IP F-URH49 prot_score	FLAG-IP F-UAP56 prot_score
A0A024R341	NIF3L1BP1	17845	73	31
A0A075B6K9	IGLC2	11341	0	44
A0A075B719	CEP152	66989	30	0
A0A075B730	EPPK1	552791	216	0
A0A075B7D9	TAF15	61793	69	131
A0A087WT95	ACOT2	50898	32	0
A0A087WTT1	PABPC1	58499	175	0
A0A087WUL9	PSMD13	42712	33	0
A0A087WVQ6	CLTC	191936	31	0
A0A087WVZ9	POLR2E	21446	51	0
A0A087WW66	PSMD1	105784	166	0
A0A087WWE2	POLR2A	218067	0	31
A0A087WWS1	THOC1	75605	398	1148
A0A087WWU8	TPM3	26404	59	63
A0A087WX84	AKAP9	190620	74	85
A0A087WYS6	PSMA8	25025	0	32
A0A087X1X7	EEF1D	69240	74	0
A0A087X210	P2RX3	44148	36	35
A0A087X2D0	SRSF3	10314	53	0
A0A087X2I1	PSMC6	45768	54	0
A0A0A0MRQ5	PRDX1	10670	100	93
A0A0B4J231	IGLL5	23136	0	1
A0A0B4J259	LYZ	15319	0	35
A0A0B4J2C3	TPT1	22559	32	0
A0A0C4DG98	THOC2	182659	360	1412
A0A0G2JIW1	HSPA1B	70066	404	11
A0A0G2JLD8	SSBP1	15588	46	60
A0A0G2JNU3	BDP1	293719	31	31
A0A0G2JPF8	HNRNPCL4	32038	250	0
A0A0R4J2E8	MATR3	94565	119	45
A0A1B0GV23	CTSD	43803	129	194
A6NHQ2	FBLL1	34782	72	0
A6NKF1	SAC3D1	43526	0	282
B1AN99	PRSS3	19276	0	55
B1APP6	PFKP	26054	36	0
B3KT61	RALYL	31055	51	0
B4DJC3	H2AFY	21534	91	0
B4DUR8	CCT3	55639	32	0
C9JAK5	ARF4	17688	49	0

C9K0J5	RAPH1	141091	30	0
D6REC9	THOC3	38747	250	345
E5RG95	ENO3	16447	104	0
E5RHP0	NME1	17138	87	0
E7EPB6	CFTR	163250	0	1
E7EQB2	LTF	76577	0	338
E7EQR4	EZR	69329	77	0
E7EUU4	EIF4G1	171535	53	0
E7EWB4	DPYSL5	20754	38	0
E9PB61	ALYREF	27541	0	508
E9PBF6	LMNB1	66368	147	313
E9PEQ6	PPIE	27681	50	0
E9PKE3	HSPA8	68763	568	113
F5GWF6	CCT2	56771	62	0
F5H793	LDHB	10800	40	0
F8VR77	PA2G4	31447	49	0
F8VVM2	SLC25A3	36138	35	0
F8WAR6	KIF3C	75783	0	34
F8WCP5	THOC5	78458	309	901
F8WE65	PPIA	13013	171	0
G3V1A4	CFL1	16801	40	0
G3V3R6	LGALS3	24071	76	0
H0Y532	SELENBP1	33903	59	0
H0Y5H9	SERPINB4	42472	0	43
H0Y8G5	HNRNPD	29649	101	0
H0YB24	CCAR2	68881	64	0
H0YGG5	A2ML1	110496	48	0
H0YH81	ATP5B	38226	117	0
H0YHC3	NAP1L1	23403	165	0
H0YIB4	SRSF9	12717	0	32
H0YKS4	ANXA2	38580	209	358
H0YKT8	PSMA4	19949	0	57
H0YL72	IDH3A	35763	41	0
H3BSM5	GABARAPL2	10395	41	0
H7BZ35	DARS	21594	0	30
H7BZK5	GGCT	20994	97	137
H7C4E7	CSDC2	16853	30	0
I3L0K7	TRAP1	57184	93	0
I3L246	RPS15A	27150	46	0
I3L397	EIF5A	16009	41	0
J3KP15	SRSF2	15362	39	0
J3KQC6	TMPRSS13	63127	3	5
J3KRG2	GSDMA	49334	62	98
J3QQM1	PSMC5	29328	66	0
J3QS13	PSMD11	10075	44	0

J3QS39	UBB	10463	228	0
K7EJP1	ATP5A1	59714	225	0
K7EK07	H3F3B	14905	207	0
K7EL20	EIF3G	29265	44	0
K7ES02	BLMH	37174	0	187
M0QXL5	FBL	26623	130	0
M0R132	KLK10	15726	48	42
O43823	AKAP8	76061	69	0
O60884	DNAJA2	45717	32	0
O75648	TRMU	47714	0	1
O94906	PRPF6	106858	34	0
O95197	RTN3	112541	33	0
O95347	SMC2	135572	31	0
O95400	CD2BP2	37623	34	0
O95816	BAG2	23757	232	0
O96019	ACTL6A	47430	47	0
P01213	PDYN	28367	34	0
P01876	IGHA1	37631	0	56
P02545	LMNA	74095	392	0
P04040	CAT	59719	0	41
P04792	HSPB1	22768	105	0
P05089	ARG1	34713	0	248
P06702	S100A9	13234	38	76
P06733	ENO1	47139	0	220
P06748	NPM1	32555	48	0
P07305	H1F0	20850	47	0
P07900	HSP90AA1	84607	354	0
P08238	HSP90AB1	83212	314	0
P0C0S5	H2AFZ	13545	114	0
P0C0S8	HIST1H2AG	14083	220	163
P10412	HIST1H1E	21852	219	0
P10809	HSPD1	61016	55	0
P11021	HSPA5	72288	1255	0
P12236	SLC25A6	32845	142	0
P12270	TPR	267131	0	32
P14174	MIF	12468	33	0
P16401	HIST1H1B	22566	140	0
P17066	HSPA6	70984	228	0
P22735	TGM1	89730	95	309
P23490	LOR	25744	0	38
P25311	AZGP1	34237	59	0
P26641	EEF1G	50087	93	0
P28838	LAP3	56131	235	0
P29508	SERPINB3	44537	89	0
P30041	PRDX6	25019	74	0

P31025	LCN1	19238	0	89
P31689	DNAJA1	44839	37	0
P31942	HNRNPH3	36903	20	2
P31946	YWHAB	28065	65	1
P31947	SFN	27757	246	1
P32119	PRDX2	21878	260	140
P32929	CTH	44479	32	0
P35998	PSMC2	48603	46	0
P36952	SERPINB5	42073	140	0
P38646	HSPA9	73635	126	45
P42357	HAL	72652	0	545
P43686	PSMC4	47337	149	0
P46821	MAP1B	270468	73	0
P47929	LGALS7	15066	255	40
P48163	ME1	64109	0	46
P51178	PLCD1	85611	36	0
P52597	HNRNPF	45643	296	0
P55060	CSE1L	110346	205	31
P60842	EIF4A1	46125	211	0
P62191	PSMC1	49154	140	0
P62318	SNRPD3	13907	57	52
P68366	TUBA4A	49892	344	0
Q02413	DSG1	113676	452	322
Q03252	LMNB2	69906	49	93
Q07021	C1QBP	31343	0	101
Q07283	TCHH	253777	0	36
Q08188	TGM3	76584	331	434
Q08554	DSC1	99924	331	165
Q09161	NCBP1	91781	0	41
Q09666	AHNAK	628699	171	28
Q12874	SF3A3	58812	34	0
Q13061	TRDN	81546	41	0
Q13200	PSMD2	100136	101	0
Q13263	TRIM28	88493	272	0
Q13509	TUBB3	88324	356	0
Q13835	PKP1	82808	339	0
Q15233	NONO	54197	42	0
Q15293	RCN1	38866	124	0
Q15365	PCBP1	37474	37	0
Q15424	SAFB	102580	0	423
Q15828	CST6	16500	86	0
Q16610	ECM1	60635	0	61
Q1KMD3	HNRNPUL2	85052	0	32
Q2L6G8	CDSN	51507	67	55
Q53F19	NCBP3	70549	0	503

Q53FA3	HSPA1L	70360	430	0
Q53RT3	ASPRV1	36968	75	78
Q5H8X8	UTS2	16266	41	0
Q5JP01	RBBP7	31483	42	0
Q5JVF3	PCID2	46000	0	155
Q5QPL9	RALY	24650	167	0
Q5VU13	VSIG8	43863	85	0
Q6E0U4	DMKN	47054	46	0
Q6I9Y2	THOC7	23728	258	516
Q6MZM0	HEPHL1	131519	86	0
Q6ZVX7	NCCRP1	30828	42	97
Q86SJ6	DSG4	113751	227	0
Q86W42	THOC6	37511	257	732
Q86Y56	DNAAF5	93462	35	0
Q8NEA5	C19orf18	24136	0	31
Q8TF66	LRRC15	64325	184	0
Q8TF72	SHROOM3	216724	11	0
Q8WVV4	POF1B	68022	226	84
Q8WXQ8	CPA5	49005	31	0
Q96FQ6	S100A16	11794	0	64
Q96M42	LINC00479	15199	31	31
Q96P63	SERPINB12	46247	41	79
Q9BVA1	TUBB2B	49921	148	0
Q9BXP5	SRRT	100604	0	507
Q9NPP4	NLRC4	116085	35	34
Q9NTU7	CBLN4	21794	31	0
Q9NYB0	TERF2IP	44233	58	0
Q9UJY1	HSPB8	21591	59	0
Q9ULC6	PADI1	74618	53	0
Q9Y446	PKP3	87029	227	0
Q9Y617	PSAT1	40397	90	0

Supplementary Table 2-1. List of detected peptide fragment by limited proteolysis from UAP56ΔN42

pep_#	pep_start	pep_end	pep_seq	A1	A2	A3	A4
1	49	58	DFLLKPELLR	1.45	2.03	1.83	1.88
2	124	131	ELAFQISK	1.33	1.36	1.32	1.66
3	139	144	YMPNVK	1.36	1.32	1.13	1.25
4	139	155	YMPNVKVAVFFGGLSIK	2.05	2.71	3.97	1.88
5	145	155	VAVFFGGLSIK	1.72	2.09	0.9	1.45
6	145	156	VAVFFGGLSIKK	2.05	2.4	1.99	1.88
7	156	162	KDEEVLK	2.05	1.6	1.39	2.07
8	157	162	DEEVLK	3.07	2.71	2.98	3.29
9	163	175	KNCPHIVVGTPGR	0.82	0.72	2.32	1.25
10	164	175	NCPHIVVGTPGR	1.18	1.33	0.88	1.18
11	176	181	ILALAR	1.42	1.33	1.51	1.77
12	184	188	SLNLK	2.05	5.41	0.79	0.75
13	192	200	HFILDECDK	1.67	1.64	1.37	1.53
14	192	208	HFILDECDKMLEQLDMR	2.56	2.71	1.32	1.67
15	201	208	MLEQLDMR	1.46	1.42	1.18	1.6
16	209	216	RDVQEIFR	1.64	1.44	1.24	1.64
17	210	216	DVQEIFR	1.16	1.75	1.89	1.74
18	223	233	QVMMFSATLSK	1.7	1.35	1.52	1.05
19	241	256	KFMQDPMEIFVDDETK	0.97	1.23	1.54	1.46
20	242	256	FMQDPMEIFVDDETK	1.25	1.4	1.27	1.36
21	242	268	FMQDPMEIFVDDETKLTLHGLQQYYVK	2.05	0.9	1.99	1.88
22	257	268	LTLHGLQQYYVK	1.47	1.18	1.55	1.17
23	269	274	LKDNEK	0.68	0	1.99	0
24	300	319	CIALAQLLVEQNFPAIAIHR	1.9	0	0.66	0
25	320	326	GMPQEER	1.49	0	1.16	0
26	339	349	RILVATNLFGR	0	0	0	0
27	340	349	ILVATNLFGR	0.07	0.06	0.09	0.04
28	350	355	GMDIER	0	0	0	0
29	381	398	FGTKGLAITFVSDENDAK	0	0	0	0
30	385	398	GLAITFVSDENDAK	0	0	0	0
31	399	406	ILNDVQDR	0	0	0	0
32	399	428	ILNDVQDRFEVNISELPDEIDISSYIEQTR	0	0	0	0
33	407	428	FEVNISELPDEIDISSYIEQTR	0	0	0	0

Supplementary Table 2-2. List of detected peptide fragment by limited proteolysis from URH49ΔN41

pep_#	pep_start	pep_end	pep_seq	R1	R2	R3	R4
34	48	57	DFLLKPELLR	1.26	0.08	1.27	1.62
35	123	130	ELAFQISK	1.72	0.38	1.43	1.79
36	138	143	YMPSVK	0.63	0.17	1.04	0.87
37	138	154	YMPSVKVSVFFGGLSIK	1.3	0	1.83	2.45
38	144	154	VSVFFGGLSIK	1.56	0.45	1.12	1.36
39	144	155	VSVFFGGLSIKK	5.2	0.9	1.83	2.3
40	155	161	KDEEVLK	3.9	2.7	1.83	3.68
41	156	161	DEEVLK	1.73	0.3	3.65	3.15
42	162	174	KNCPHVVVGTPGR	1.3	0	0.91	1.23
43	163	174	NCPHVVVGTPGR	1.8	0.31	1.2	1.34
44	175	180	ILALVR	1.77	0.31	1.51	1.72
45	183	187	SFSLK	3.9	0	0.91	1.84
46	191	199	HFVLDECDK	0.62	0.22	1.03	1.2
47	191	207	HFVLDECDKMLEQLDMR	1.56	0.27	1.26	1.98
48	200	207	MLEQLDMR	0.81	0.19	1.24	1.25
49	208	215	RDVQEIFR	1.17	0	1.33	1.51
50	209	215	DVQEIFR	2.4	0.25	1.83	1.84
51	222	232	QCMMFSATLSK	1.3	0.3	2.28	2.3
52	240	255	KFMQDPMEVFVDDETK	0.5	1.32	1.3	1.05
53	241	255	FMQDPMEVFVDDETK	0.74	0.56	1.14	1.1
54	241	267	FMQDPMEVFVDDETKLTLHGLQQYYVK	1.95	1.35	1.83	3.68
55	256	267	LTLHGLQQYYVK	0.67	1.52	1.2	1.09
56	268	273	LKDSEK	1.95	2.25	1.83	0
57	299	318	CMALAQLLVEQNFPAIAIHR	1.67	1.54	1.26	0
58	319	325	GMAQEER	0.62	1.42	1.04	0
59	338	348	RILVATNLFGR	0.19	2.57	0	0
60	339	348	ILVATNLFGR	0.09	1.74	0.09	0.09
61	349	354	GMDIER	0.49	3.04	0	0
62	380	397	FGTKGLAITFVSDENDAK	0	2.7	0	0
63	384	397	GLAITFVSDENDAK	0.11	2.78	0	0
64	398	405	ILNDVQDR	0.22	3	0.06	0
65	398	427	ILNDVQDRFEVNVAELPEEIDISTYIEQSR	0	3.78	0	0
66	406	427	FEVNVAELPEEIDISTYIEQSR	0.14	1	0	0

Supplementary Table 3. List of amino acid residues with significantly different conformations

1XTI			Fr48			Difference of conformation
Amino acid residue	Dihedral angle	Conformation class	Amino acid residue	Dihedral angle	Conformation class	
89A	psi	0	88A	psi	3	3
90K	phi	1	89K	phi	4	3
112G	phi	1	111G	phi	4	3
149F	psi	3	148F	psi	0	3
151G	phi	2	150G	phi	5	3
**245D**	phi	1	**244D**	phi	4	3
**254E**	psi	2	**253E**	psi	5	3
**257L**	psi	3	**256L**	psi	0	3
287F	psi	3	286F	psi	0	3
336F	phi	1	335F	phi	4	3
346L	phi	4	345L	phi	1	3
**346L**	psi	4	**345L**	psi	1	3
**350G**	phi	1	**349G**	phi	4	3
**350G**	psi	3	**349G**	psi	0	3
**354E**	psi	0	**353E**	psi	3	3
382G	phi	5	381G	phi	2	3
383T	psi	0	382T	psi	3	3
407F	psi	0	406F	psi	3	3

Supplementary Table 4. Antibodies list in this study

Name	Sorce	Identifier
Mouse monoclonal anti-FLAG (M2)	Sigma-Aldrich	F1804
Rabbit polyclonal anti-β-actin	Sigma-Aldrich	A2066
Rabbit polyclonal anti-HNRNPM	Sigma-Aldrich	HPA024344
Mouse monoclonal anti-SRRM2	Sigma-Aldrich	S4045
Mouse monoclonal anti-HA (12CA5)	GeneTex	GTX16918
Mouse polyclonal anti-GAPDH	FUJIFILM Wako	016-25523
Rabbit polyclonal anti-THOC1	10.1101/gad.1302205	NA
Rabbit polyclonal anti-THOC2	10.1101/gad.1302205	NA
Rabbit polyclonal anti-THOC5	10.1101/gad.1302205	NA
Rabbit polyclonal anti-ALYREF	10.1101/gad.1302205	NA
Rat polyclonal anti-CIP29	10.1091/mbc.E09-10-0913	NA
Rat polyclonal anti-UAP56	10.1091/mbc.E09-10-0913	NA
Rat polyclonal anti-URH49	10.1091/mbc.E09-10-0913	NA
Rat polyclonal anti-RUVBL1	This paper	NA
Rat polyclonal anti-RUVBL2	This paper	NA
Rat polyclonal anti-ILF2	This paper	NA
Rat polyclonal anti-ILF3	This paper	NA

Supplementary Table 5. Primer list for plasmid construction used in this study

Name		Nucleotide sequence (5' → 3')	plasmid constructions
KpnI-UAP56-CDS start	Forward	ACTCGGTACCATGGCAGAGAACGATGTGGA	pcDNA5-3xFLAG-UAP56, pGEX6p2-UAP56
UAP56-CDS end-XhoI	Reverse	CCGCTCGAGCTACCGTGTCTGTTCAATGT	
KpnI-URH49-CDS start	Forward	CTACTCGGTACCATGGCAGAACAGGATGTG	pcDNA5-3xFLAG-URH49, pGEX6p2-URH49
URH49-CDS end-XhoI	Reverse	CCGCTCGAGTTACCGGCTCTGCTCGATGT	
KpnI-CIP29-CDS start	Forward	ACTCGGTACCATGGCGACCGAGACGGTGGA	pcDNA5-3xFLAG-CIP29, pcDNA5--HA-CIP29
CIP29-CDS end-XhoI	Reverse	CCGCTCGAGTCAGGCAATCCCAAAGCGCT	
BamH1-RUVBL1-CDS start	Forward	CTGGGATCCATGAAGATTGAGGAGGTG	pcDNA5-3xFLAG-RUVBL1
RUVBL1-CDS end-XhoI	Reverse	CGGCTCGAGTCACTTCATGTACTTATCCTG	
BamH1-RUVBL2-CDS start	Forward	CAAGGATCCATGGCAACCGTTAC	pcDNA5-3xFLAG-RUVBL2
RUVBL2-CDS end-XhoI	Reverse	CAACTCGAGTCAGGAGGTGTCCAT	
KpnI-ILF2-CDS start	Forward	TGACAAGCTTGGTACCATGCGTCCAATGCG	pcDNA5-3xFLAG-ILF2
ILF2-CDS end-XhoI	Reverse	GCCCTCTAGACTCGAGTCACTCCTGAG	
Infusion-ILF3-CDS start	Forward	TGACAAGCTTGGTACCATGCGTCCAATGCG	pcDNA5-3xFLAG-ILF3
ILF3-CDS end-Infusion	Reverse	GCCCTCTAGACTCGAGGATATCTTATCTGTAC	
Infusion-HNRNPM-CDS start	Forward	CACAAAGCCCAGACGCGGAG	pcDNA5-3xFLAG-HNRNPM
HNRNPM-CDS end-Infusion	Reverse	GGCAACTGCTTAAGCGTTTCTATC	
BamHI-RUVBL1_for_antigen	Forward	AATGGATCCTTGCCAAAAGGGGATGTGC	pMALc2x-RUVBL1 (250-456 amino acids)
RUVBL1_for_antigen-HindIII	Reverse	GCGAAGCTTTCACTTCATGTACTTATCCTG	
BamHI-RUVBL2_for_antigen	Forward	TCATTTACGTGAAGGAGGAGGAGG	pMALc2x-RUVBL2 (1-225 amino acids)
RUVBL2_for_antigen-HindIII	Reverse	TATGGATCCCACGAACTTGGTCTG	
EcoRI-ILF2_for_antigen	Forward	AATGAATTCACACCCTGGATCCTTG	pMALc2x-ILF2 (240-390 amino acids)
ILF2_for_antigen-HindIII	Reverse	AATAAGCTTTCACTCCTGAGTTTCCATGC	
BamHI-ILF3_for_antigen	Forward	CGCGGATCCGAGAAAGTATTAGCTGGAG	pMALc2x-ILF3 (280-355 amino acids)
ILF3_for_antigen-HindIII	Reverse	CGCAAGCTTGTAGTCCACTGGG	
UAP56 siRNA-resinstant mutagenesis-F	Forward	TAGTGAGCTGCCAGACGAAATTGATATAAGTAG TTACATTGAACA	pcDNA5-3xFLAG-UAP56 (siR)
UAP56 siRNA-resinstant mutagenesis-F	Forward	TGTTCAATGTAACTACTTATATCAATTTCGTCTG GCAGCTCACTA	
URH49 siRNA-resinstant mutagenesis-F	Forward	GTGCTGGCCACATTGCAACAAATCGAACCAGT GAACGGACAGGTG	pcDNA5-3xFLAG-URH49 (siR)
URH49 siRNA-resinstant mutagenesis-F	Forward	CACCTGTCCGTTCACTGGTTCGATTTGTTGCA ATGTGGCCAGCAC	

UAP56 K95N mutagenesis-F	Forward	TCGGGCATGGGAAATACAGCAGTGTTTGTC	pcDNA5-3xFLAG-UAP56 K95N
UAP56 K95N mutagenesis-R	Reverse	GACAAACACTGCTGTATTTCCCATGCCCGA	
URH49 K94N mutagenesis-F	Forward	TCCGGGATGGGCAACACAGCGGTCTTCGTC	pcDNA5-3xFLAG-URH49 K94N
URH49 K94N mutagenesis-R	Reverse	GACGAAGACCGCTGTGTTGCCCATCCCGGA	
URH49 I106L mutagenesis-F	Forward	GCCACATTGCAACAACTTGAACCAGTGAAC	pcDNA5-3xFLAG-URH49 I106L
URH49 I106L mutagenesis-R	Reverse	GTTCACTGGTTCAAGTTGTTGCAATGTGGC	
URH49 N110T mutagenesis-F	Forward	CAAATCGAACCAGTGACCGGACAGGTGACG	pcDNA5-3xFLAG-URH49 N110T
URH49 N110T mutagenesis-R	Reverse	CGTCACCTGTCCGGTCACTGGTTCGATTTG	
URH49 S141N mutagenesis-F	Forward	TCCAAGTACATGCCCAACGTCAAGGTGTCT	pcDNA5-3xFLAG-URH49 S141N
URH49 S141N mutagenesis-R	Reverse	AGACACCTTGACGTTGGGCATGTACTTGGA	
URH49 S145A mutagenesis-F	Forward	CCCAGCGTCAAGGTGGCGTGTTCTTCGGT	pcDNA5-3xFLAG-URH49 S145A
URH49 S145A mutagenesis-R	Reverse	ACCGAAGAACACAGCCACCTTGACGCTGGG	
URH49 V167I mutagenesis-F	Forward	AAGAACTGTCCCCATATCGTGGTGGGGACC	pcDNA5-3xFLAG-URH49 V167I
URH49 V167I mutagenesis-R	Reverse	GGTCCCCACCACGATATGGGGACAGTTCTT	
URH49 V179A mutagenesis-F	Forward	CGCATCCTGGCGCTCGCGCGGAATAGGAGC	pcDNA5-3xFLAG-URH49 V179A
URH49 V179A mutagenesis-R	Reverse	GCTCCTATTCCGCGCGAGCGCCAGGATGCG	
URH49 R182K mutagenesis-F	Forward	GCGCTCGTGCGGAATAAGAGCTTCAGCCTA	pcDNA5-3xFLAG-URH49 R182K
URH49 R182K mutagenesis-R	Reverse	TAGGCTGAAGCTCTTATTCCGCACGAGCGC	
URH49 N188H mutagenesis-F	Forward	AGCTTCAGCCTAAAGCATGTGAAGCACTTT	pcDNA5-3xFLAG-URH49 N188H
URH49 N188H mutagenesis-R	Reverse	AAAGTGCTTCACATGCTTTAGGCTGAAGCT	
URH49 V189I mutagenesis-F	Forward	TTCAGCCTAAAGAATATAAAGCACTTTGTG	pcDNA5-3xFLAG-URH49 V189I
URH49 V189I mutagenesis-R	Reverse	CACAAAGTGCTTTATATTCTTTAGGCTGAA	
URH49 V193I mutagenesis-F	Forward	ATGTGAAGCACTTTTATACTGGACGAGTGT	pcDNA5-3xFLAG-URH49 V193I
URH49 V193I mutagenesis-R	Reverse	ACACTCGTCCAGTATAAAAGTGCTTCACAT	
URH49 L216M mutagenesis-F	Forward	CAGGAGATCTTCCGCATGACACCACACGAG	pcDNA5-3xFLAG-URH49 L216M
URH49 L216M mutagenesis-R	Reverse	CTCGTGTGGTGTCATGCGGAAGATCTCCTG	
URH49 C223V mutagenesis-F	Forward	CCACACGAGAAGCAGGTCATGATGTTGAGC	pcDNA5-3xFLAG-URH49 C223V
URH49 C223V mutagenesis-R	Reverse	GCTGAACATCATGACCTGCTTCTCGTGTGG	
UAP56 V224C mutagenesis-F	Forward	CACGAGAAGCAGTGCATGATGTTCAGTGCT	pcDNA5-3xFLAG-UAP56 V224C
UAP56 V224C mutagenesis-R	Reverse	AGCACTGAAACATCATGCACTGCTTCTCGTG	
UAP56 183-186 mutagenesis-F	Forward	GCTCGAAATAGGAGCTTCAGCCTCAAACAC	pcDNA5-3xFLAG-UAP56 183-186MUT
UAP56 183-186 mutagenesis-R	Reverse	GTGTTTGAGGCTGAAGCTCCTATTTCGAGC	
URH49 182-185 mutagenesis-F	Forward	TGCGGAATAAGAGCTTGAACCTAAAGAATG	pcDNA5-3xFLAG-URH49 182-185MUT
URH49 182-185 mutagenesis-R	Reverse	CATTCTTTAGGTTCAAGCTCTTATTCCGCA	

UAP56 Nter chimera mutagenesis-F	Forward	CCATCCACAGCTCTGGCTTCCGGGACTTTC	pcDNA5-3xFLAG-UAP56 N-term chimera
UAP56 Nter chimera mutagenesis-R	Reverse	GAAAGTCCCGGAAGCCAGAGCTGTGGATGG	
UAP56 Cter chimera mutagenesis-F	Forward	CGCTTTGAGGTCAATGTGGCAGAACTTCCA	pcDNA5-3xFLAG-UAP56 C-term chimera
UAP56 Cter chimera mutagenesis-R	Reverse	TGGAAGTTCTGCCACATTGACCTCAAAGCG	
URH49 Nter chimera mutagenesis-F	Forward	CCATCCACAGCTCTGGCTTTCGTGACTTCC	pcDNA5-3xFLAG-URH49 N-term chimera
URH49 Nter chimera mutagenesis-R	Reverse	GGAAGTCACGAAAGCCAGAGCTGTGGATGG	
URH49 Cter chimera mutagenesis-F	Forward	CGGTTTGAAGTTAATATTAGTGAGCTGCCA	pcDNA5-3xFLAG-URH49 C-term chimera
URH49 Cter chimera mutagenesis-R	Reverse	TGGCAGCTCACTAATATTAACTTCAAACCG	
UAP56_URH49 core chimera mutagenesis-F	Forward	ACGCTGCATGGGTTGCAGCAGTACTACGT	pcDNA5-3xFLAG-UAP56 N-core/C-core chimera, pcDNA5-3xFLAG-URH49 N-core/C-core chimera
UAP56_URH49 core chimera mutagenesis-R	Reverse	ACGTAGTACTGCTGCAACCCATGCAGCGT	
BamHI-UAP56-CDS start	Forward	GTGATTGGATCCATGGCAGAGAACGATGTG	pGEX6p2-UAP56
EcoRI-URH49-CDS start	Forward	CCATGAATTCCCATGGCAGAACAGGATGTG	pGEX6p2-URH49
BamHI-UAP56ΔN42	Forward	CGCGGATCCATGCACAGCTCTGGCTTTCGT	pGEX6p2-UAP56ΔN42
EcoRI-URH49ΔN41	Forward	CGCGAATTCCCATGCACAGCTCTGGCTTCC	pGEX6p2-URH49ΔN41
BamHI-SUB2Δ59	Forward	ACTCGGATCCCATTCCACCGGTTTCAAA	pGEX6p2-SUB2Δ59
SUB2-CDS end-XhoI	Reverse	CCGCTCGAGTTAATTATTCAAATAAGTGGA	

Supplementary Table 6. siRNA list used in this study

Name		Nucleotide sequence (d:deoxyribonucleotide)	Supplier	Source
EGFP	sense(5'-3')	GGGCACAAGCUGGAGUACAACUACA	invitrogen/stealth	10.1091/mbc.E09-10-0913
(Control)	antisense(5'-3')	UGUAGUUGUACUCCAGCUUGUGCCC	invitrogen/stealth	
UAP56	sense(5'-3')	CCUGAUGAGAUAGACAUCUCCUCCU	invitrogen/stealth	10.1091/mbc.E09-10-0913
	antisense(5'-3')	AGGAGGAGAUGUCUAUCUCAUCAGG	invitrogen/stealth	
URH49	sense(5'-3')	CCCUACAGCAGAUUGAGCCUGUCAA	invitrogen/stealth	10.1091/mbc.E09-10-0913
	antisense(5'-3')	UUGACAGGCUCAAUCUGCUGUAGGG	invitrogen/stealth	
CIP29 #1	sense(5'-3')	CAAAGCCCAUUGAGCUCCCUGUCAA	invitrogen/stealth	10.1091/mbc.E09-10-0913
	antisense(5'-3')	UUGACAGGGAGCUCAAUGGGCUUUG	invitrogen/stealth	
RUVBL1 #1	sense(5'-3')	CCACAGAGAUGGAGACCAUCUACdGdA	IDT	This Study
	antisense(5'-3')	UCGUAGAUGGUCUCCAUCUCUGUGGUC		
ILF3 #1	sense(5'-3')	GCGGAUCCGACUACAACUACGAGdAdG	IDT	10.1016/j.bbrc.2012.07.103
	antisense(5'-3')	CUCUCGUAGUUGUAGUCGGAUCCGCCC		
hnRNPM #1	sense(5'-3')	ACAUCGAUACGAGACCUCUGAAUdTdT	IDT	This Study
	antisense(5'-3')	AAAUUCAGAGGUCUCGUAUCGAUGUUU		
CIP29 #2	sense(5'-3')	GCUUUCUUACUCUCCAAGCUCACAGGU	IDT	10.1016/j.bbagrm.2020.194480
	antisense(5'-3')	CUGUGAGCUUGGAGAGUAAGAAAdGdC		
RUVBL1 #2	sense(5'-3')	GUUUACUCAACUGAGAUCAAGAAdGdA	IDT	10.1016/j.molcel.2017.01.019.
	antisense(5'-3')	UCUUCUUGAUCUCAGUUGAGUAAACUU		
ILF3 #2	sense(5'-3')	CUACGAGAGCAAAUUCAACUACAdGdT	IDT	10.1128/MCB.00120-08
	antisense(5'-3')	ACUGUAGUUGAAUUUGCUCUCGUAGUU		
hnRNPM #2	sense(5'-3')	GUUGUUGAAUUCAAGAUGGAAGAdGdA	IDT	This Study
	antisense(5'-3')	GACAACAACUUAAGUUCUACCUUCUCU		

Supplementary Table 7. Primer list for RT-qPCR and RT-PCR used in this study

Name		Nucleotide sequence	Source
PGK1	Forward	GTTGCAGACAAGATCCAGCTC	10.1091/mbc.E09-10-0913
	Reverse	GAAGTGCCAATCRCCATGTTG	
PRC1	Forward	GATTGAGGCAATTCGAGTGGAGCTGG	10.1091/mbc.E09-10-0913
	Reverse	AGCATCGTGGAGCTGGAGCAGAC	
CENPA	Forward	TATTGGCCCTACAAGAGGCAG	This Study
	Reverse	GGCCAGTTGCACATCCTTTG	
MCM2	Forward	AGAATCTATGGCGACAGGCAG	This Study
	Reverse	ATAGTCCCGCAGATGGATGC	
CHEK1	Forward	TCATGGCAGGGGTGGTTTATC	This Study
	Reverse	TTGCCAAGCCAAAGTCTGAG	
MT2A	Forward	CCCGCTCCCAGATGTAAAG	This Study
	Reverse	GGAATATAGCAAACGGTCACG	
HN1	Forward	TCAGCAGGTGCCAAGTCTAGTG	This Study
	Reverse	CTGCCTGGCAAGTCTGTGT	
PLP2	Forward	TGGCCCTGGAGTGATTTCTTC	This Study
	Reverse	CCTGCGACGATTTTGGAGTG	
tRNA	Forward	GTAGTCGTGGCCGAGTGGTTAAG	10.1016/j.isci.2019.11.033
	Reverse	GTAGTCGGCAGGATTCGAACC	
U6 snRNA	Forward	GTGCTCCCTTCGGCAAGCACTTACACTAAA	10.1016/j.isci.2019.11.033
	Reverse	CTCAAAAAGGAATGCTTCACAAATTTGCCT	
E2F8	Forward	CGGGGAGGAGAATAAGTACG	10.1016/j.isci.2019.11.033
	Reverse	CTTGTCTTTGCGGCTGTTTA	
