# Supplementary material for: Structural differences between the closely related RNA helicases, UAP56 and URH49, fashion distinct functional apo-complexes
Source: Nat Commun. 2024 Jan 15;15:455. doi: 10.1038/s41467-023-44217-8 (PMC10789772; doi:10.1038/s41467-023-44217-8)
Supplement: Supplementary file 3 — Reporting Summary [file 41467_2023_44217_MOESM3_ESM.pdf]

nature portfolio

Corresponding author(s): Ken-ichi Fujita
Seiji Masuda

Last updated by author(s): October 13, 2023

Reporting Summary

Nature Portfolio wishes to improve the reproducibility of the work that we publish. This form provides structure for consistency and transparency in reporting. For further information on Nature Portfolio policies, see our Editorial Policies and the Editorial Policy Checklist.

Statistics

For all statistical analyses, confirm that the following items are present in the figure legend, table legend, main text, or Methods section.

n/a	Confirmed	
☐	☒	The exact sample size (*n*) for each experimental group/condition, given as a discrete number and unit of measurement
☐	☒	A statement on whether measurements were taken from distinct samples or whether the same sample was measured repeatedly
☐	☒	The statistical test(s) used AND whether they are one- or two-sided<br>*Only common tests should be described solely by name; describe more complex techniques in the Methods section.*
☐	☒	A description of all covariates tested
☐	☒	A description of any assumptions or corrections, such as tests of normality and adjustment for multiple comparisons
☐	☒	A full description of the statistical parameters including central tendency (e.g. means) or other basic estimates (e.g. regression coefficient) AND variation (e.g. standard deviation) or associated estimates of uncertainty (e.g. confidence intervals)
☐	☒	For null hypothesis testing, the test statistic (e.g. *F*, *t*, *r*) with confidence intervals, effect sizes, degrees of freedom and *P* value noted<br>*Give P values as exact values whenever suitable.*
☒	☐	For Bayesian analysis, information on the choice of priors and Markov chain Monte Carlo settings
☒	☐	For hierarchical and complex designs, identification of the appropriate level for tests and full reporting of outcomes
☒	☐	Estimates of effect sizes (e.g. Cohen's *d*, Pearson's *r*), indicating how they were calculated

Our web collection on statistics for biologists contains articles on many of the points above.

Software and code

Policy information about availability of computer code

Data collection	The diffraction images of URH49ΔN41 crystal were collected at 100 K (in a cold nitrogen gas stream) on a Rayonix MX225 CCD detector (Rayonix, Evanston, IL) with a wavelength of 0.9 Å at BL26B1 in SPring-8 (Hyogo, Japan).
Data analysis	Gene ontology (GO) was analyzed using Database for Annotation, Visualization and Integrated Discovery (DAVID: version 6.7)<br>The diffraction images sets of URH49ΔN41 crystal were processed, merged, and scaled using XDS (version Mar. 31, 2022). The structure was solved by molecular replacement with UAP56ΔN42 (Protein Data Bank entry 1XTI) using a search mode by Molrep implemented in CCP4i software (version 7.0.073). The model was refined using PHENIX 1.20.1 software, rebuilt using COOT 0.8.9. Protein structure images were depicted using PyMOL software (The PyMOL Molecular Graphics System, Version 2.0 Schrödinger, LLC).<br>MD simulation was performed for the apo-form of URH49ΔN41 using Desmond Molecular Dynamics System, version 5.2 (D. E. Shaw Research, New York, NY).

For manuscripts utilizing custom algorithms or software that are central to the research but not yet described in published literature, software must be made available to editors and reviewers. We strongly encourage code deposition in a community repository (e.g. GitHub). See the Nature Portfolio guidelines for submitting code & software for further information.

Data

Policy information about availability of data

All manuscripts must include a data availability statement. This statement should provide the following information, where applicable:

- Accession codes, unique identifiers, or web links for publicly available datasets
- A description of any restrictions on data availability
- For clinical datasets or third party data, please ensure that the statement adheres to our policy

Atomic coordinates and structure factors for the reported crystal structures have been deposited with the Protein Data bank under accession number 8IJU (URH49Δ41).

Research involving human participants, their data, or biological material

Policy information about studies with human participants or human data. See also policy information about sex, gender (identity/presentation), and sexual orientation and race, ethnicity and racism.

Reporting on sex and gender	N/A
Reporting on race, ethnicity, or other socially relevant groupings	N/A
Population characteristics	N/A
Recruitment	N/A
Ethics oversight	N/A

Note that full information on the approval of the study protocol must also be provided in the manuscript.

Field-specific reporting

Please select the one below that is the best fit for your research. If you are not sure, read the appropriate sections before making your selection.

☒ Life sciences ☐ Behavioural & social sciences ☐ Ecological, evolutionary & environmental sciences

For a reference copy of the document with all sections, see nature.com/documents/nr-reporting-summary-flat.pdf

Life sciences study design

All studies must disclose on these points even when the disclosure is negative.

Sample size	The sample size of each experiment is described in the figure legends in the main manuscript and supplementary file.
Data exclusions	No data was excluded from this study.
Replication	The replication number is indicated in the legend of corresponding figures, where applicable.
Randomization	All the control and experimental group of cells were grown under identical conditions. Sample was takn at random.
Blinding	All the control and experimental group of cells were grown under identical conditions. Blinding was used to all samples.

Reporting for specific materials, systems and methods

We require information from authors about some types of materials, experimental systems and methods used in many studies. Here, indicate whether each material, system or method listed is relevant to your study. If you are not sure if a list item applies to your research, read the appropriate section before selecting a response.

Materials & experimental systems

n/a	Involved in the study
☐	☒ Antibodies
☐	☒ Eukaryotic cell lines
☒	☐ Palaeontology and archaeology
☐	☒ Animals and other organisms
☒	☐ Clinical data
☒	☐ Dual use research of concern
☒	☐ Plants

Methods

n/a	Involved in the study
☒	☐ ChIP-seq
☒	☐ Flow cytometry
☒	☐ MRI-based neuroimaging

Antibodies

Antibodies used

Antibodies were obtained as follows: FLAG M2 mouse monoclonal antibody (1:3000 dilution; F1804, Sigma-Aldrich Japan, Tokyo, Japan), rabbit anti-β-actin antibody (1:3000 dilution; A2066, Sigma-Aldrich Japan), rabbit anti-HNRNPM antibody (1:2000 dilution; HPA024344, Sigma-Aldrich Japan) and mouse anti-SRRM2 antibody (1:2000 dilution; S4045, Sigma-Aldrich Japan), HA (12CA5) mouse monoclonal antibody (1:2000 dilution; GTX16918, GeneTex, Irvine, CA), mouse anti-GAPDH antibody (1:2000 dilution; 016-25523, Fujifilm Wako), sera against THOC1 (1:1000 dilution), THOC2 (1:1000 dilution), THOC5 (1:1000 dilution), ALYREF (1:1000 dilution), CIP29 (1:1000 dilution), UAP56 (1:1000 dilution) and URH49 (1:1000 dilution) have been described previously11. Anti-RUVBL1 (1:1000 dilution), anti-RUVBL2 (1:1000 dilution), anti-ILF2 (1:1000 dilution), and anti-ILF3 sera (1:1000 dilution) were prepared from immunized rats as described previously in accordance with the recommendations in the Guide for the Care and Use of Laboratory Animals of the Animal Committee in Kyoto University (Animal experiments were approved by the Committee on the Ethics of Animal Experiments of Kyoto University, Experiment permission number: Lif-K17002). The antibodies used in this study are listed in supplemental Table 4.

Validation

All antibodies purchased from commercial companies have been validated by the companies as described below.

FLAG M2 mouse monoclonal antibody (1:3000 dilution; F1804, Sigma-Aldrich Japan, Tokyo, Japan)
Information of this antibody
(website) https://www.sigmaaldrich.com/JP/ja/product/sigma/f1804
(validation) chrome-extension://efaidnbmnnnibpcajpcglclefindmkaj/https://www.sigmaaldrich.com/deepweb/assets/sigmaaldrich/product/documents/754/849/anti-flag-2poster.pdf
(reference) Roquin binds microRNA-146a and Argonaute2 to regulate microRNA homeostasis. Monika Srivastava et al. Nature communications, 6, 6253-6253 (2015-02-24), https://www.nature.com/articles/ncomms7253,
We used anti-FLAG M2 mouse monoclonal antibody in Fig.1B. Source data are provided as a Source Data file.

rabbit anti-β-actin antibody (1:3000 dilution; A2066, Sigma-Aldrich Japan)
(website & validation) https://www.sigmaaldrich.com/JP/ja/product/sigma/a2066
(reference) Effects of doxorubicin cancer therapy on autophagy and the ubiquitin-proteasome system in long-term cultured adult rat cardiomyocytes. Dimitrakis P, et al. Cell and Tissue Research, 350(2), 361-372 (2012), https://link.springer.com/article/10.1007/s00441-012-1475-8
We used rabbit anti-β-actin antibody in Supplementary Fig.3A. Source data are provided as a Source Data file.

rabbit anti-HNRNPM antibody (1:2000 dilution; HPA024344, Sigma-Aldrich Japan)
(website & validation) https://www.sigmaaldrich.com/JP/ja/product/sigma/hpa024344
(reference) Identification of HnRNP M as a novel biomarker for colorectal carcinoma by quantitative proteomics. Chen S, et al. American Journal of Physiology: Gastrointestinal and Liver Physiology, 306(5), 394-403 (2014) https://journals.physiology.org/doi/full/10.1152/ajpgi.00328.2013?rfr_dat=cr_pub++0pubmed&url_ver=Z39.88-2003&rfr_id=ori%3Arid%3Acrossref.org
We used rabbit anti-HNRNPM antibody in Fig.1D. Source data are provided as a Source Data file.

mouse anti-SRRM2 antibody (1:2000 dilution; S4045, Sigma-Aldrich Japan)
(website & validation) https://www.sigmaaldrich.com/JP/ja/product/sigma/s4045
(reference) Paraquat modulates alternative pre-mRNA splicing by modifying the intracellular distribution of SRPK2. Silvia Vivarelli et al. PloS one, 8(4), e61980-e61980 (2013-04-25) https://journals.plos.org/plosone/article?id=10.1371/journal.pone.0061980
We used mouse anti-SRRM2 antibody in Fig.2C. Source data are provided as a Source Data file.

HA (12CA5) mouse monoclonal antibody (1:2000 dilution; GTX16918, GeneTex, Irvine, CA)
(website & validation) https://www.genetex.com/Product/Detail/HA-tag-antibody-12CA5/GTX16918
(reference) Dephosphorylation and genome-wide association of Maf1 with Pol III-transcribed genes during repression. Roberts DN et al. Mol Cell 2006; 22 (5):633-44 https://www.sciencedirect.com/science/article/pii/S1097276506002541?via%3Dihub#fig2
We used HA (12CA5) mouse monoclonal antibody in Supplementary Fig.1C. Source data are provided as a Source Data file.

mouse anti-GAPDH antibody (1:2000 dilution; 016-25523, Fujifilm Wako)
(website) https://labchem-wako.fujifilm.com/jp/product/detail/W01W0101-2552.html
(reference) Altered sulfation status of FAM20C-dependent chondroitin sulfate is associated with osteosclerotic bone dysplasia Toshiyasu Koike, et al. Nat Commun. 2022; 13: 7952. https://www.ncbi.nlm.nih.gov/pmc/articles/PMC9792594/
We used mouse anti-GAPDH antibody in Supplementary Fig.3A Source data are provided as a Source Data file.

Antibodies against THOC1, THOC2, THOC5, ALYREF, CIP29, UAP56 and URH49 have been described previously(10.1016/j.bbagrm.2020.194480).

Anti-RUVBL1, anti-RUVBL2, anti-ILF2, and anti-ILF3 sera were validated in this study.

Eukaryotic cell lines

Policy information about cell lines and Sex and Gender in Research

Cell line source(s)	U2OS was obtained from ATCC (HTB-96), MCF7, and A549 cells were obtained from JCRB (JCRB0134, JCRB0076), Flp-In T-REx 293 cell was obtained from Thermo Fisher Scientific (Waltham, MA)
Authentication	None
Mycoplasma contamination	These cell lines were not contaminated with mycoplasma.
Commonly misidentified lines (See ICLAC register)	None

Animals and other research organisms

Policy information about studies involving animals; ARRIVE guidelines recommended for reporting animal research, and Sex and Gender in Research

Laboratory animals	Wistar rat, 8 weeks of female,
Wild animals	No wild animals were used.
Reporting on sex	Wistar rats (female) were used to prepare antisera. These antisera were used for the detection of RUVBL1 (1:1000 dilution), anti-RUVBL2 (1:1000 dilution), anti-ILF2 (1:1000 dilution), and anti-ILF3 (1:1000 dilution). This is why we lacked sex- and gender-based analysis.
Field-collected samples	This study did not involve samples collected from the field.
Ethics oversight	Part of this study included the animal experiment to prepare Anti-RUVBL1, anti-RUVBL2, anti-ILF2, and anti-ILF3 sera prepared from immunized rats in accordance with the recommendations in the Guide for the Care and Use of Laboratory Animals of the Animal Committee in Kyoto University (Animal experiments were approved by the Committee on the Ethics of Animal Experiments of Kyoto University, Experiment permission number: Lif-K17002).

Note that full information on the approval of the study protocol must also be provided in the manuscript.

April 2023
